# The standardisation of the approach to metagenomic human gut analysis: from sample collection to microbiome profiling

**DOI:** 10.1038/s41598-022-12037-3

**Published:** 2022-05-19

**Authors:** Natalia Szóstak, Agata Szymanek, Jan Havránek, Katarzyna Tomela, Magdalena Rakoczy, Anna Samelak-Czajka, Marcin Schmidt, Marek Figlerowicz, Jan Majta, Kaja Milanowska-Zabel, Luiza Handschuh, Anna Philips

**Affiliations:** 1 Institute of Bioorganic Chemistry, Polish Academy of Sciences, 61-704 Poznan, Poland; 2 Ardigen S.A., ul. Podole 7, 30-394 Cracow, Poland; 3 Department of Cancer Immunology, Chair of Medical Biotechnology, Poznan University of Medical Sciences, 60-806 Poznan, Poland; 4 Department of Food Biotechnology and Microbiology, Poznan University of Life Sciences, 60-627 Poznan, Poland

**Keywords:** Microbial communities, Microbiology techniques

## Abstract

In recent years, the number of metagenomic studies increased significantly. Wide range of factors, including the tremendous community complexity and variability, is contributing to the challenge in reliable microbiome community profiling. Many approaches have been proposed to overcome these problems making hardly possible to compare results of different studies. The significant differences between procedures used in metagenomic research are reflected in a variation of the obtained results. This calls for the need for standardisation of the procedure, to reduce the confounding factors originating from DNA isolation, sequencing and bioinformatics analyses in order to ensure that the differences in microbiome composition are of a true biological origin. Although the best practices for metagenomics studies have been the topic of several publications and the main aim of the International Human Microbiome Standard (IHMS) project, standardisation of the procedure for generating and analysing metagenomic data is still far from being achieved. To highlight the difficulties in the standardisation of metagenomics methods, we thoroughly examined each step of the analysis of the human gut microbiome. We tested the DNA isolation procedure, preparation of NGS libraries for next-generation sequencing, and bioinformatics analysis, aimed at identifying microbial taxa. We showed that the homogenisation time is the leading factor impacting sample diversity, with the recommendation for a shorter homogenisation time (10 min). Ten minutes of homogenisation allows for better reflection of the bacteria gram-positive/gram-negative ratio, and the obtained results are the least heterogenous in terms of beta-diversity of samples microbial composition. Besides increasing the homogenisation time, we observed further potential impact of the library preparation kit on the gut microbiome profiling. Moreover, our analysis revealed that the choice of the library preparation kit influences the reproducibility of the results, which is an important factor that has to be taken into account in every experiment. In this study, a tagmentation-based kit allowed for obtaining the most reproducible results. We also considered the choice of the computational tool for determining the composition of intestinal microbiota, with Kraken2/Bracken pipeline outperforming MetaPhlAn2 in our in silico experiments. The design of an experiment and a detailed establishment of an experimental protocol may have a serious impact on determining the taxonomic profile of the intestinal microbiome community. Results of our experiment can be helpful for a wide range of studies that aim to better understand the role of the gut microbiome, as well as for clinical purposes.

## Introduction

The human body is inhabited by a vast number of microorganisms; it is estimated that the microbes in a healthy human adult at least equal the number of human cells^1^. Among them, bacteria emerged as the key players. Although it has long been known that these microorganisms are not only passers-by but are also involved in processes such as food digestion and vitamin production, recent evidence has shown that the microbiome has a much broader impact, with the gut microbiome being of particular interest. From diabetes^2^ and obesity^3^ to the possible impact on autism spectrum disorder^4,5^ and other mental diseases, successive studies are slowly revealing a bidirectional relationship between gut microorganisms and human health.

The field of microbiome research is one of the most dynamically growing fields among biomedical sciences. Although the scientific community was aware of commensal bacteria’s role in health and wellbeing, the next-generation sequencing (NGS) revolution of the 2000s finally enabled a wider view of the microbiome structure. The metagenomic approach that complemented traditional, culture-based studies allowed for unprecedented insight into the populations of microorganisms inhabiting various ecological niches, such as the human gut. This momentum may be observed in the exponential growth of published papers on the microbiome in recent decades, an increase from several papers in the 1980s to over 25,000 in 2021 (Supplementary Fig. 1).

The most famous project dedicated to understanding the human body microbiome is the National Institutes of Health (NIH) Human Microbiome Project^6^. The first phase of this project characterized the microbiomes of healthy humans at five major body sites using 16S and metagenomic shotgun sequencing. The second phase integrated datasets from both the microbiome and the host collected within three different cohort studies of microbiome-associated conditions (pregnancy and preterm birth, onset of inflammatory bowel disease, onset of type 2 diabetes) using multiple omics technologies. Another significant ongoing study is the Microbiome Signature Project, which is a Swedish-Danish Microbiome initiative that aims to enhance cross-border and cross-disciplinary collaborations and synergies, establish international research facilities and activities and attract international talents to publicly and privately funded research organizations. This initiative concentrates on researching how the microbiome influences human health with the aim of using the microbiome in the fight against diseases such as asthma, diarrhoea and obesity. Moreover, there are many smaller scientific projects devoted to the study of the human microbiome and its impact on human health^7–11^.

Although the best practices for metagenomics studies have been the topics of several publications^12–14^ and the main aim of the International Human Microbiome Standard (IHMS) project, standardisation of procedure for analysing metagenomic data is still far from being achieved. Despite the increasing number of metagenomic studies, a standard procedure for analysing metagenomic data is still under debate. Given the tremendous complexity and variability of microbial communities, the problem with standardisation results in the batch effect, which may cause incompatibility of various datasets produced even in projects coming from the same study and/or laboratory^15,16^. Comparing results obtained by multiple researchers (which is a standard approach in human genomics) is hardly possible in metagenomic studies. Taking this into account, as well as the increasing amount of data generated in metagenomic experiments and the impact of the results on medicine, establishing the standards for such experiments is a must. Only with such an approach is it possible to provide reproducibility of results and to leverage the potential of metagenomic studies that will give rise to trustworthy insights and effective novel therapies.

Each experimental procedure should be seen as a detailed algorithm, which means that it should be carefully designed and strictly followed. While thinking of metagenomic experiments, no step is negligible: starting with sample collection through DNA isolation, sequencing, and various bioinformatics analyses to statistical testing. Each part of the procedure is crucial for obtaining reliable results that lead to meaningful conclusions.

Taking into consideration the still up-to-date problems with the standardisation of the procedure for analysing the microbial samples and the need to establish this kind of procedure, we decided to carefully examine each stage of such analyses. Our sample collection procedure is fully complement with IHMS_SOP 05 V2^17^. However, the IHMS provides no protocol for samples’ DNA extraction stabilised according to IHMS_SOP 05 V2. Therefore we have developed our protocol based on DNeasy PowerSoil Pro Kit (Qiagen) recommended in Human Microbiome Project Core Microbiome Sampling Protocol A, HMP Protocol no. 07-001^18^. Our protocol combines sample collection and stabilisation recommended by IHMS with samples DNA Extraction recommended by HMP. Based on that, we aim to provide a step-by-step practical guidance approach for metagenomic gut microbiome studies. For this purpose, we thoroughly analysed the procedure of DNA isolation, preparation of NGS libraries, and bioinformatics analysis aimed at identifying gut microbial taxa.

## Methods

### Aim of the study

In this study, we intend to establish a standard approach to investigating gut metagenomes. We checked how experimental procedures on different stages of the analyses influenced community profiling and investigated the bias caused by subsequent bioinformatics steps.

### Experiment design

**Figure 1:**
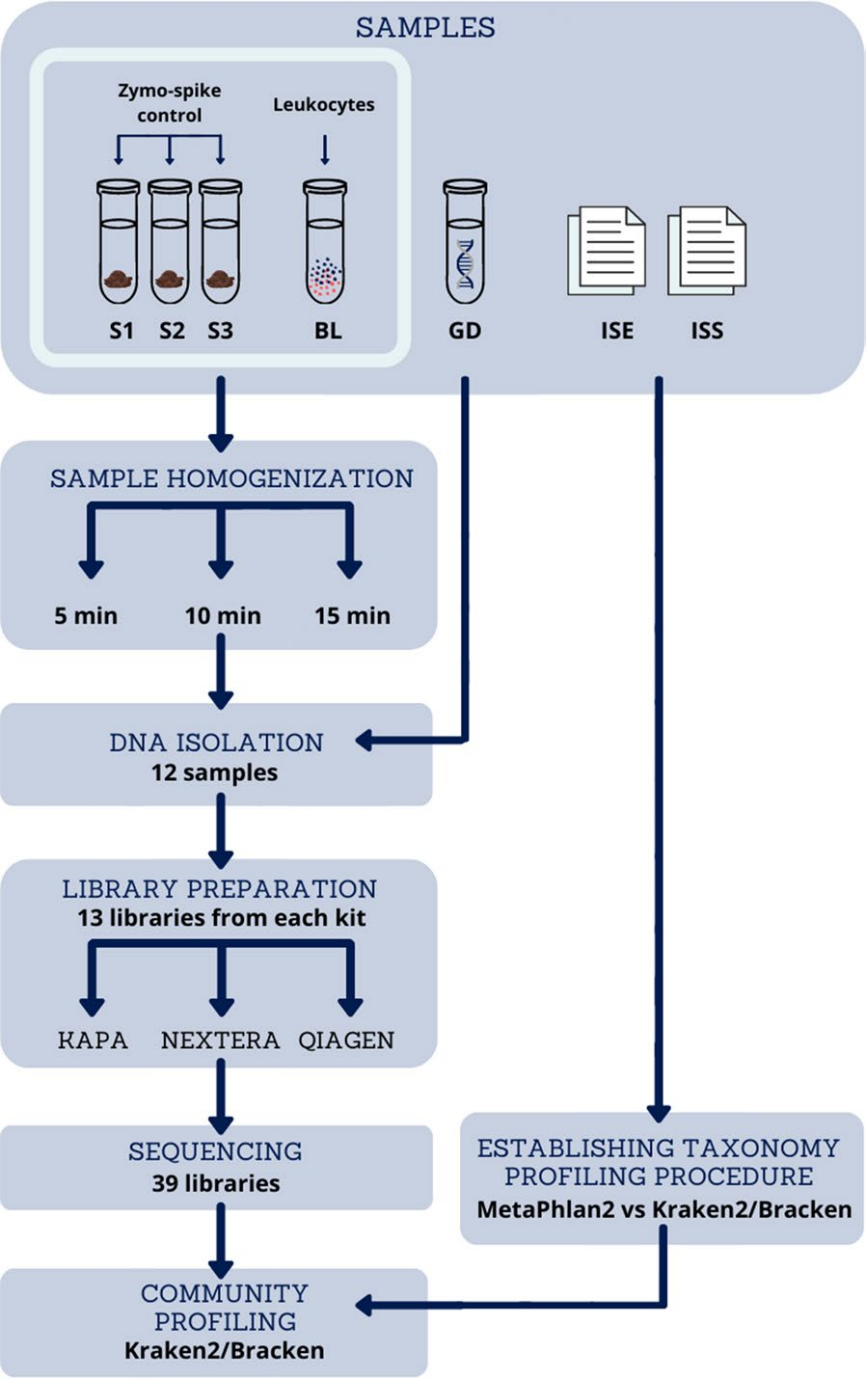
Overview of the experiment design. *S1, S2, S3* samples from volunteers, *BL* ATCC bacterial mix, *GD* ATCC genomic DNA, *ISE, ISS* simulated NGS samples (ISE—even species abundance distribution, ISS—log-normal species abundance distribution).

We designed a test experiment that allowed us to scrutinize each stage of the analysis of the intestinal metagenomes (Fig. 1). We started with stool sampling, followed by DNA isolation and NGS library preparation, and finished with computational methods for microbial community profiling.

For this study, we used (i) three faecal samples obtained from healthy volunteers (S1, S2, S3), (ii) two commercially available bacterial mixes (ATCC Bacterial Mix MSA-2006—a mix of 12 known bacteria (BL) and ATCC Genomic DNA MSA-1003—a mix of 20 bacterial DNAs (GD)) and (iii) two in silico-generated samples reflecting the composition of ATCC Bacterial Mix (ISE, ISS). Both ATCC controls come from authenticated mock microbial communities selected based on relevant phenotypic and genotypic attributes, such as Gram staining, GC content, genome size, and spore formation, thus mimicking mixed metagenomic samples. For details, see the “Samples” section.

All methods were carried out in accordance with relevant guidelines and regulations.

### Samples, homogenisation and DNA isolation

To test the impact of the DNA extraction procedure on microbial profiling, we examined three real human faecal samples (S1, S2, and S3) and a sample prepared from ATCC Bacterial Mix (ATCC^®^ MSA-2006™, further referred as BL). The bacterial composition of ATCC Bacterial Mix is known and served as a reference in our study. Moreover, the three volunteer samples were enriched with so-called Zymo-spikes: two bacterial strains (gram-positive *Allobacillus halotolerans* and gram-negative *Imtechella halotolerans*) that are normally not present in human intestines. Zymo-spikes were added to investigate the differences in gram-negative and gram-positive bacterial DNA extraction efficiency, library preparation, and subsequent abundance profiling, as the two bacterial types differ significantly in their cell envelope structure. Gram-negative bacteria have a thin peptidoglycan cell wall sandwiched between an inner cytoplasmic cell membrane and a bacterial outer membrane, whereas gram-positive bacteria characterize a much thicker, multi-layered peptidoglycan cell wall and lack of an outer membrane. These differences may cause significant bias in the DNA isolation procedure. Moreover, as host cells are also abundant in stool^19^, we added human peripheral blood mononuclear cells (PBMCs) to ATCC Bacterial Mix to investigate whether there is a preference during DNA isolation toward human or bacterial DNA. The samples were then subjected to homogenisation for three different times tested: 10, 15 and 20 min.

### Library preparation and sequencing

As a next step, we prepared DNA libraries using three sets of reagents from leading manufacturers—KAPA, Illumina (Nextera) and Qiagen, which were selected based on their popularity and differences in the DNA fragmentation procedure. In total, we obtained 36 libraries. Additionally, we introduced a new sample at this step, ATCC Genomic Mix DNA isolate (ATCC^®^ MSA-1003™, further referred as GD), to serve as an isolation reference, giving three additional libraries (hereinafter referred to as KAPA, Nextera, and Qiagen). In total, we prepared 39 libraries—13 libraries with each kit. All libraries were then subjected to DNA sequencing (Table 1).

**Table 1:** IDs of sequencing libraries prepared for analyses.

Source of samples	ZYMO spike									ATCC bacterial mix + human leukocytes (–BL)			ATCC genomic DNA (–GD)
	S1 (–S1)			S2 (–S2)			S3 (–S3)						
Bead Beating time (in min)	10 (–1–)	15 (–2–)	20 (–3–)	10	15	20	10	15	20	10	15	20	(NA)
Kappa (K–)	K1S1	K2S1	K3S1	K1S2	K2S2	K3S2	K1S3	K2S3	K3S3	K1BL	K2BL	K3BL	KGD
Nextera (N–)	N1S1	N2S1	N3S1	N1S2	N2S2	N3S2	N1S3	N2S3	N3S3	N1BL	N2BL	N3BL	NGD
Qiagen (Q–)	Q1S1	Q2S1	Q3S1	Q1S2	Q2S2	Q3S2	Q1S3	Q2S3	Q3S3	Q1BL	Q2BL	Q3BL	QGD

Simultaneously, with DNA sequencing, we prepared two in silico samples that contained a mix of reads derived from genomes of bacteria constituting the ATCC bacterial mix (ISE and ISS). One simulated sample reflected the bacterial abundance of the ATCC bacterial mix, while for the other, we sampled the abundances from the log-normal distribution. These two simulated samples served as controls to test bioinformatics tools used for the analysis of bacterial community composition.

### Bioinformatics processing

Apart from analysing samples generated in the course of the experiment, we processed the in silico simulated samples with two leading profiling tools: MetaPhlAn2^20^ and Kraken2/Bracken combination^21,22^. MetaPhlAn2 is based on the mapping of predefined microbial marker sequences, while Kraken is based on matching *k*-mers obtained from a sequencing read with the database of *k*-mers derived from the database of microbial genomes. We additionally investigated the content of human DNA in the sequenced samples using read mapping against the human genome^23^ with BWA MEM^24^. Similarly, we mapped the reads against the reference genomes of Zymo-spike species to compare the coverage of reads mapping to those genomes and inferred gram-negative and gram-positive species isolation bias. With the metrics recall, weighted precision, root mean square error (RMSE) of abundance, and beta diversity, we assessed the methods in terms of community reconstruction efficiency.

### Samples

#### Volunteers’ samples (S1, S2, S3)

Stool samples were collected from three donors—S1, S2, S3 and are a part of the Polish Microbiome Map project (ClinicalTrials.gov study identifier: NCT04169867). The biological material was stabilised immediately after collection using RNAlater Stabilisation Solution (Invitrogen, Thermo Fisher Scientific); see Sampling and Storage sections.

To S1, S2, S3 samples, 25 µL of ZymoBIOMICS™ Spike-in Control I (High Microbial Load, Catalog No. D6320) was added to estimate gram-positive and gram-negative species DNA isolation efficiency. These two bacterial species (*Allobacillus halotolerans* and *Imtechella halotolerans*) are absent in the human gut.

#### ATCC bacterial mix with leucocytes (BL)

Gut Microbiome Whole Cell Mix (ATCC^®^ MSA-2006™) is a mixture of 12 bacterial strains that are typical of the human gut microbiome (Supplementary Table 1). To this sample, approximately 10,000 human PBMCs were added to simulate host cells that are normally present in stool samples^25^. Those samples are hereafter referred to as BL. PBMCs were isolated from volunteers' whole blood by standard gradient centrifugation in Histopaque 1077 (Sigma-Aldrich). Freshly isolated cells were counted in Türk’s solution, and 10,000 cells were added to the bacterial mix.

#### ATCC genomic DNA (GD)

Gut Microbiome Genomic Mix (ATCC^®^ MSA-1003™) is a commercially available mixture of genomic DNA from 20 bacterial strains typical of the human gut microbiome; for short, we will further refer to those samples as GD.

#### Simulated NGS samples (ISE, ISS)

To establish the taxonomy assignment procedure, we used artificial read sets generated with CAMISIM^26^, a tool for simulating shotgun metagenomic data. We prepared two samples of approximately 1 million reads each (Supplementary Table 2). Both samples contained the same species as the BL sample, but the first reflected the mixture in terms of even species abundance (ISE), while the second had staggered abundances sampled from a log-normal distribution (ISS) (Supplementary Table 3). At the level of reads, we used an ART simulator^27^ (ART-MountRainier-2016-06-05) with an MBARC-26 error profile^28^, which is the duo dedicated for modelling Illumina sequencing of bacterial communities.

### Sampling and storing volunteer samples

Faecal samples (approx. 1 g) were self-collected by three donors into vials containing 3 mL of RNAlater Stabilisation Solution (Invitrogen, ThermoFisher Scientific) and delivered by courier within 24 h to the laboratory, where the samples were anonymized and stored at 4 °C for up to 1 week. Each person provided signed informed consent for participating in the study. Appropriate approval was also obtained from the Bioethical Commission of the Karol Marcinkowski University of Medical Sciences (resolution No. 485/19, passed on 11th April 2019). Each sample was homogenised to reduce possible differences in the spatial distribution of microbial cells in faecal mass by manual stirring with a spatula, and afterwards split into three tubes. Tubes were centrifuged at 14,000*g* for 5 min, the supernatant was discarded, and residues were transferred to a − 20 °C freezer for storage until DNA extraction (two weeks).

### DNA isolation

The frozen stool samples were thawed on ice, and DNA was extracted from them using a DNAeasy PowerSoil Pro Kit (Qiagen, Germany) according to the manufacturer's instructions with the following protocol adjustments. The liquid phase of stabilised stool samples (excess of RNAlater Stabilisation Solution) was separated by centrifugation at 10,000*g* for 3 min and thoroughly discarded to remove high salt content that may interfere with a subsequent DNA purification step. Next, the stabilised stool and fresh BL samples (250 mg) were bead-beaten in PowerBead Pro tubes containing proprietary beads using a Mixer Mill MM400 (Retsch, Germany) for 10, 15 or 20 min at 25 Hz. Each sample was injected with 5 µL RNase (10 mg/mL concentration; A&A Biotechnology, Poland) and incubated at 60 °C for 10 min to allow RNA digestion. This step removes RNA allowing to increase DNA yield. The DNA quality was verified with agarose gel electrophoresis. The final DNA concentration was measured by a Nanodrop ND-1000 spectrophotometer (Thermo Fisher Scientific, USA) (Supplementary Table 4). All the differences in the extracted DNA amount may be due to the nonhomogeneous nature of the stool sample material.

### DNA library preparation

Libraries were constructed with the following commercial kits: (1) KAPA HyperPlus (Roche, Switzerland); (2) Nextera DNA Flex Library Prep (now under the new name, Illumina DNA Prep, Illumina, USA); and (3) QIAseq FX DNA Library Kit (Qiagen, Germany), according to the manufacturer’s protocols (Supplementary Table 5). 500 ng of stool-extracted DNA was used for each library preparation. Different parameters of tagmentation/enzymatic fragmentation reaction were set for each kit to aim at the 400–500 bp average fragment size of libraries for 500 ng DNA input. The time range was adjusted according to the manufacturer’s protocols to desired library fragment size. In the library amplification step, six PCR cycles were applied. Library concentration was measured using a Qubit fluorometer and Qubit DNA HS Assay Kit (Thermo Fisher Scientific, USA).

Purified libraries were stored for up to 2 weeks at − 20 °C until sequencing. The quality of libraries and fragment distribution were analysed using a Bioanalyzer 2100 and DNA 1000 Kit or High Sensitivity DNA Kit (Agilent Technologies, USA), depending on the obtained library quantity. This quality control of purified libraries was performed up to one week before the sequencing run.

### Next generation sequencing

Prior to sequencing, all libraries were thawed on ice and normalized to the final 10 nM concentration. Thirty-nine different libraries with distinctive index combinations were pooled together and diluted with EB Buffer (Qiagen, Germany) to obtain a mix of 2 nM libraries, according to Protocol A: Standard Normalization Method for the NextSeq system (Illumina, USA). Sequencing was performed with NextSeq 550 (Illumina, USA) using High Output Kit v2.5 reagents (Illumina, USA); approximately 10 million 150 bp paired-end reads were generated per library.

### Data preprocessing and quality control

In the first stage of raw data processing, we ran demultiplexing on the raw BCL intensity file with the bcl2fastq tool^29^ for base calling and separating the reads from different samples. Moreover, bcl2fastq generated a barcode summary report, which allowed us to track back the origin of the undetermined barcodes in terms of the index source kit. To assess the quality of the sequencing procedure, we generated quality control reports with FastQC^30^ and MultiQC^31^. We preprocessed the raw fastq reads with cutadapt^32^ using the following procedure: we trimmed the adapter sequences (based on TruSeq adapter sequences) and poly-G tails observed in the data, which are characteristic of the two-channel sequencing technology of NextSeq. We also filtered out reads shorter than 140 bases to remove the bias in taxonomy profiling that could emerge from the shorter sequences. The remaining reads were subjected to further analysis.

### Mapping to the human genome

To determine human-derived contamination and assess the fraction of human reads in the studied samples, we mapped filtered reads to the GRCh38.p12 version of the human reference genome with the BWA MEM^24^ aligner.

### Calibration of bioinformatics taxonomy profiling methods

#### Tools for community composition profiling

To examine the impact of a tool on bacterial community profiling results, we compared two leading programs that employ completely different strategies: Kraken2^21^ and MetaPhlAn2^20^. Both tools were run with default settings.

Kraken2 is a classification system that uses exact matches of the *k*-mers from the query sequence to the lowest common ancestor of all the genomes in the database holding this *k*-mer to inform the classification algorithm. Bracken^22^ is an accompanying tool of Kraken2 that is used to obtain a quantitative profile of the samples. Bracken employs probabilistic re-estimation of taxa abundance based on Kraken’s read-level taxonomy assignment. For taxonomy assignment with Kraken2, we used a full Kraken2 database provided by the developers of the tool (the version downloaded on 6th October 2019).

MetaPhlAn2^20^ employs a clade-specific marker gene database to reconstruct a qualitative and quantitative profile of the sample community. The database of the marker genes for MetaPhlAn2 was derived from ~ 17,000 reference genomes from the Integrated Microbial Genomes database^33,34^ (version: mpa_v295_CHOCOPhlAn_201901).

#### Metrics used for calibration of taxonomy assignment

To obtain a quantitative measurement of how Kraken2 and MetaPhlAn2 reflect bacterial communities, we used three metrics: recall, weighted precision and RMSE. Recall is a percentage of correctly detected species in a sample (true positives, TP) compared to all species that should be detected in a given sample (true positives + false negatives, TP + FN). Weighted precision is a sum of abundances of the correctly identified species. This way, we quantify the contribution of the correctly identified species to the overall abundances within the investigated community. Traditionally, precision is defined as the percentage of correctly identified species (TP) relative to the number of all species that were detected in a given sample, correctly or not (true positives + false positives, TP + FP). However, this metric might overestimate the impact of the false positives of low abundance on the final results. This is why we used instead the weighted precision metric. The root mean square abundance error was calculated based on the absolute difference between the expected and observed species’ abundance to estimate the abundance profiling accuracy while taking into account individual species’ contribution.

Of note, species abundance reported by Bracken is relative to classified reads, and MetaPhlAn2 reports abundance relative to all of the sample input; therefore, we re-estimated MetaPhlAn2 abundances by calculating the number of reads assigned to each taxon and dividing them by the number of classified reads as a proxy for comparing those two tools.

### Bacterial communities’ analysis

#### ATCC whole cell mix and ATCC genomic DNA community reconstruction metrics

To quantify how well tested procedures allowed us to reconstruct defined communities in ATCC Whole Cell Mix (BL) and ATCC Genomic DNA samples (GD), we used the same metrics as for assessing Kraken2 and MetaPhlAn2 community reconstruction, namely, recall, weighted precision and root mean square abundance error, as described in the previous section.

#### Metrics for communities obtained with different kits and homogenisation times

We performed taxonomy profiling using Kraken2 with a confidence threshold of 0.1 and species-level quantification with Bracken. The results were assessed with methods implemented in the vegan R package^35,36^. For the purpose of comparing the same samples sequenced with different kits and homogenisation times, we calculated the Bray–Curtis dissimilarity^37^ with the mentioned package as a measure of pairwise diversity between samples. Bray–Curtis dissimilarity is defined as a proportion of the correctly identified species in both samples to the sum of all species in both samples, subtracted from 1.

#### Gram-positive and gram-negative species DNA isolation efficiency

To estimate the proportion of *Allobacillus halotolerans* and *Imtechella halotolerans* species in sequenced samples, we mapped reads against reference genomes provided by the manufacturer. For this purpose, we consistently used BWA MEM with a minimum seed length threshold of 26, with the procedure established during mapping against the human genome to increase mapping specificity. Details of this procedure and the rationale behind it are described in the Results section in the Human DNA content analysis.

As the amount of the Zymo-spikes added to the analysed samples would expectedly lead to low but consistent coverage of their genomes, with coverage peaks around regions shared between Zymo-spikes and other species in the samples, instead of directly comparing the fraction of mapped reads, we analysed the coverage along the references: for each of the two Zymo species, we analysed the regions with coverage greater than 0. We used the two following metrics: the ratio of the median abundance in covered positions and the ratio of the covered fraction of the genomes. These metrics served to assess the efficiency of gram-positive versus gram-negative species DNA isolation in volunteer samples.

We additionally assessed the gram-positive/gram-negative species’ abundance ratio in GD and BL samples, and compared it to the expected proportions. To investigate the possible impact of Gram staining status, genome length and GC content on the median species abundance across BL samples, we fit a linear model with a top-down approach as follows: we build a model including all three mentioned independent variables, with the median abundance as a dependent variable with *lm* function in R (stats R package). We iteratively removed the least significant variable from the model until we reached a final model including only variables significant for the median abundance.

#### Statistical analysis of taxonomical units for real samples

To estimate whether there are statistical differences in bacteria abundance between kits and homogenisation times at the level of clades within a given sample, we performed a pairwise comparison between different kits and different homogenisation times for each sample (S1/S2/S3). Pairwise comparisons were performed with the Wilcoxon Rank Sum test on the differences in median abundance for each tested condition (kits/homogenisation) for each sample (*compare_groups* function, metacoder R package). The results were further adjusted for multiple comparisons with the FDR method (*mutate_obs* function, metacoder R package, and *p.adjust* function, stats R package).

### Ethics approval and consent to participate

Each person provided signed informed consent for participation in the study. Appropriate approval was obtained from the Bioethical Commission of the Karol Marcinkowski University of Medical Sciences. All methods were carried out in accordance with relevant guidelines and regulations.

## Results

### DNA extraction and NGS library construction

The DNA extraction procedure was examined on the three volunteer samples (S1, S2, S3) and the BL sample. For details on volunteers’ samples and BL sample preparation, see Methods and Samples sections. Each of the S1, S2, S3 and BL samples was processed in triplicate. As presented in Fig. 1, three technical repetitions differed only in the time of mechanical sample homogenisation (5, 10 or 15 min). As a result, 12 DNA extracts were obtained. From a single stool sample (250 mg), 2–20 µg of DNA was extracted. High-quality genomic DNA with a molecular weight of approximately 20 kb was obtained from all samples (Supplementary Fig. 2). The quality of DNA was independent of the time of sample homogenisation, and only minor differences in the amount of the isolated DNA in terms of homogenisation time were observed. For the S1 and S2 samples, the extension of the homogenisation time resulted in a slight reduction in the amount of extracted DNA from the stool samples, while for the S3 samples, the amount of isolated DNA was stable, regardless of the homogenisation time. For the BL sample, the longest homogenisation time resulted in a slight increase in the amount of isolated DNA (Supplementary Table 4). To verify the correctness and efficiency of DNA extraction, we used the GD sample, which was a commercially available genomic DNA isolate from ATCC bacterial mixture.

Each kit produced libraries of different amounts and size distributions (for details, see Supplementary File 1). KAPA libraries had the longest fragments, which may suggest insufficient enzymatic fragmentation at the initial step of library preparation. Moreover, KAPA libraries had the most variable average fragment size (min 550 bp, max 1862 bp), library concentration (min 4.8 ng/µL, max 118 ng/µL) and, consequently, the final amount of library DNA (min 96.8 ng, max 2360 ng). With the Qiagen kit, the highest amounts of libraries were obtained (870–1730 ng, mean 1372.7 ng), which indicates very efficient amplification, but a higher number of PCR duplicates can be an undesirable consequence. Nextera libraries reflected the most homogeneous and repeatable results, confirming the precisely optimised fragmentation (contrary to the KAPA and Qiagen kits, which utilise enzymatic DNA fragmentation, Nextera implemented the tagmentation method) and amplification conditions in the Illumina protocol.

### Control of sequencing quality

**Figure 2:**
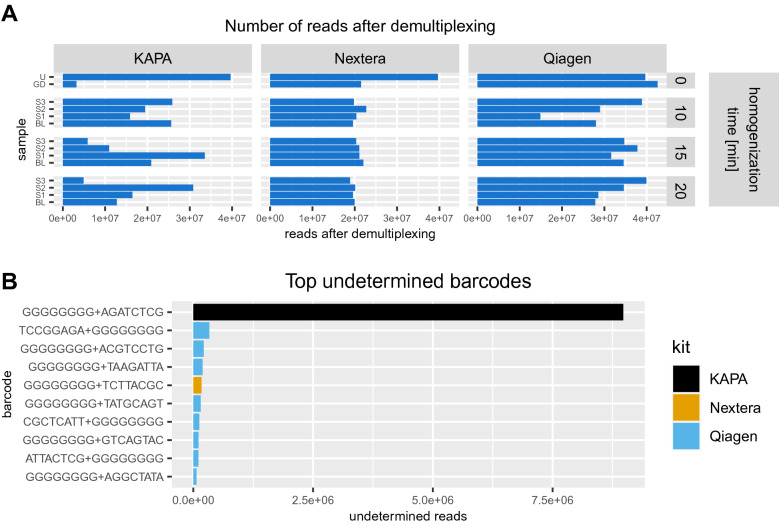
Number of reads generated for each DNA sample processed with KAPA, Nextera and Qiagen library preparation kits, following different homogenisation times: 0 min for GD mix, 10, 15 and 20 min for real samples (S1, S2, S3) and BL (**A**). The number of reads undetermined during demultiplexing was included in the plot as sample U. Number of reads with undetermined barcodes that were not successfully demultiplexed for each of the kits (**B**).

To control the quality of the resulting sequencing reads, we first compared how the number of reads was distributed across the libraries prepared with particular kits. KAPA gave the most diverse results with the lowest number of reads (16,790,404.8, std. 9,219,148.96), while Qiagen resulted in the highest number of reads (31,589,315.57, std. 7,735,638.94) but also higher variability than Nextera (20,542,238.62, std. 1,085,110.33) (Fig. 2A, Supplementary Table 6), which is consistent with our measurements of the prepared libraries. In terms of these criteria, we found Nextera to be a good compromise between the number of reads and reproducibility.

We also identified 39,651,338 reads with undetermined barcodes. We analysed the top undetermined barcodes for each kit and found that there was a significant gap between the number of undetermined reads originating from the KAPA kit and other kits (8,973,700 vs 1,476,300). Most of the undetermined barcodes had a single i5 index coming from Illumina’s TruSeq Universal Adapter sequence (Fig. 2B, Supplementary Table 7). During read preprocessing, we trimmed the adapter sequences and poly-G and filtered out reads shorter than 140 bases to remove bias in taxonomy profiling. The number of reads maintained after this treatment can be found in Supplementary Table 6.

### Establishing taxonomy profiling procedure

For the next step, we wanted to choose and calibrate a bioinformatics procedure for profiling the microbial taxonomy. We have taken into account the two leading tools that are used for taxonomy profiling: MetaPhlAn2 and Kraken2 with Bracken correction, which use orthogonal approaches to profiling the composition of the metagenomic sample. Initially, we tested both tools on the in silico samples, representing ATCC communities with an even (ISE) and staggered (ISS) distribution of species.

**Figure 3:**
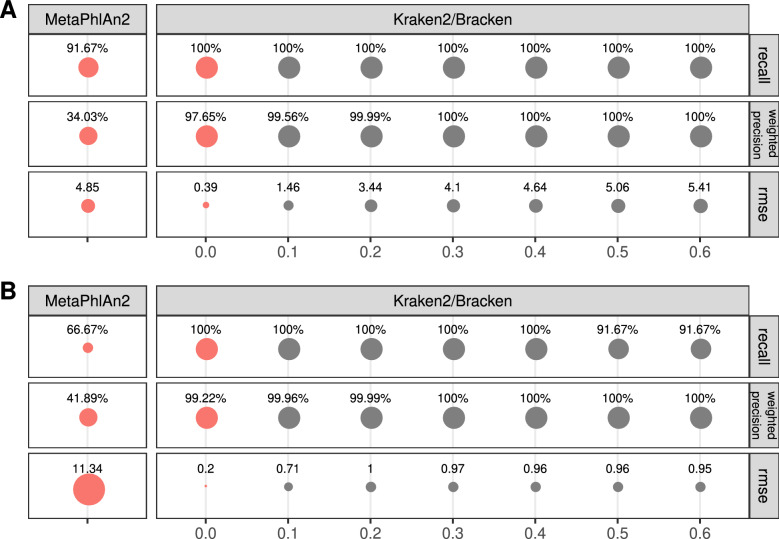
Community profiling metrics for the ISE sample (in silico even abundance) (**A**) and for the ISS sample (in silico staggered abundance) (**B**). Values for default settings of taxonomy profiling tools are highlighted in red. The Kraken2/Bracken combination identified all expected species, as opposed to MetaPhlAn2. Various Kraken2 confidence threshold values were tested to reach a higher weighted precision level and lower RMSE.

We observed that MetaPhlAn2 performs worse in terms of identifying species (recall 91.67% for even abundance and 66.67% for staggered species abundance) than the Kraken2/Bracken combination with a default confidence threshold of 0.0 (recall 100% for both even and staggered species abundance) (Fig. 3, Supplementary Table 8). The fraction of the correctly identified species in the recovered taxonomic profiles was higher for MetaPhlAn2 than for Kraken2/Bracken combination (78.57% vs 22.64% for ISS and 80% vs 64.71% for ISE, respectively). However, weighted precision for MetaPhlAn2 was much lower than for Kraken2/Bracken (34.03% for ISE and 41.89% for ISS with MetaPlAn2 vs 97.65% and 99.22% with Kraken2’s default confidence threshold). Moreover, for both samples, the RMSE of expected versus observed abundance in correctly identified species was much higher for MetaPhlAn2 profiling than for Kraken2/Bracken combination in default runs, with RMSE levels of 4.85 and 0.39, respectively, in the ISE sample with even species distribution, presenting an even larger difference for the ISS sample with a more diverse species distribution (RMSE 11.34 for MetaPhlAn2 and 0.2 for Kraken2/Bracken).

We attempted to reduce the fraction of incorrectly detected species and improve the potential of Kraken2/Bracken in community reconstruction. For this purpose, we modified the confidence threshold parameter, which is meant to improve the fidelity of community reconstruction. Therefore, we profiled the samples with Kraken2 and Bracken using different confidence threshold values, namely, 0.1, 0.2, 0.3, 0.4, 0.5, and 0.6. This parameter is expected to reduce the number of false positive results by establishing a minimum proportion of *k*-mers of a given read that have been mapped to the assigned species. An abundance summary for the default Kraken2/Bracken and MetaPhlAn2 parameters, as well as for different Kraken2 confidence thresholds, can be found in Supplementary Table 9. Results contain many species, the majority of which should not be detected in the artificial samples. Of notice, the species that were not expected were detected in a negligible abundance and only under one or two tested settings of confidence thresholds. These species should be treated as artifacts and, when considering taxonomy and its meaning, should be filtered out from the results.

Since MetaPhlAn2 was not able to detect all the expected species, showing particularly poor recall for the ISS sample, which better represents the abundance distribution expected in the real samples, we decided to use Kraken2/Bracken further in the taxonomy profiling procedure by tweaking the confidence threshold parameter. Increasing the threshold to 0.1 already brought notable improvement in terms of false species discovery, while further increasing this confidence threshold gave a slight increase in abundance error (RMSE) in the ISE sample. We decided to proceed further with 0.1 in the processing of real samples to avoid introducing additional bias in sample composition.

### Human DNA content analysis

**Figure 4:**
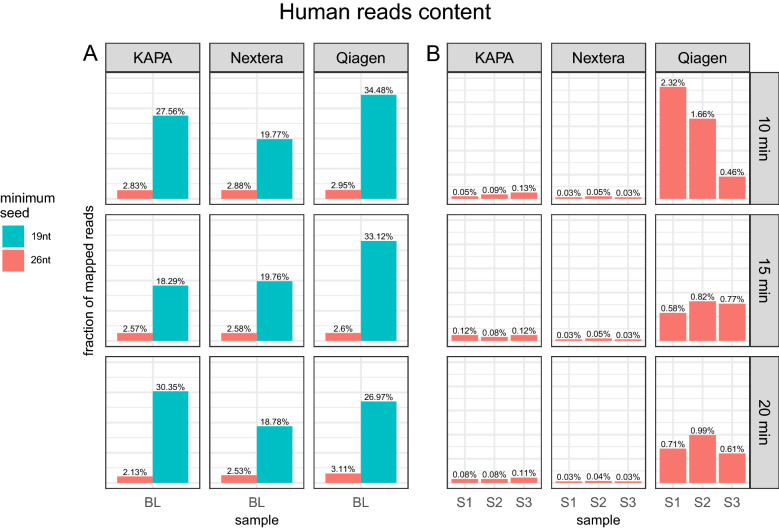
Fractions of reads mapped to the human genome in BL (**A**) with default (19 nt) and increased (26 nt) minimum BWA MEM seed length and (**B**) donor samples (minimum BWA MEM seed length 26 nt) for different kits and homogenisation times.

Read mapping (BWA MEM, default parameters, seed length of 19) against the human genome (hg38) yielded an unexpectedly high number of mapped reads (average human read fraction per sample 23.36%, standard deviation 10.38) (Fig. 4A). Interestingly, we noticed the same for the ISE and ISS in silico samples, which were generated based on only bacterial genomes. Detailed analysis revealed that the vast majority of reads that mapped to the human genome were recognized by Kraken2 as of bacterial origin, which suggests “false” mappings by BWA. Therefore, we focused on the distribution of the total number of bases along the read that mapped to the hg38 reference in the ATCC bacterial mix sample (N1BL sample spiked with human PBM cells).

**Figure 5:**
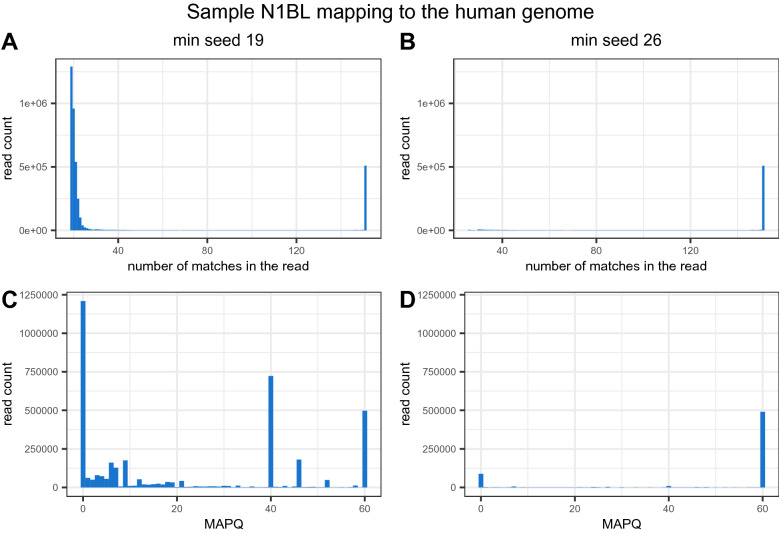
Distribution of the number of matches along the reads mapped to the human genome and corresponding mapping quality with default (**A**,**C**—19 nt) and increased BWA minimum seed length parameters (**B**,**D**—26 nt).

We observed two groups of mapped reads—one corresponding to reads mapping to the reference with a short overlap (3,139,174 reads with less than 23 nucleotides overlapping with the reference) and a large peak of 509,219 reads mapping to the reference on the entire read length. We expected that the reads with a short overlap and the reference fragment would have originated from similar regions shared by the human and bacterial species (Fig. 5A). Visualizing the mapping length distribution over the read, we also observed two peaks in the N1BL sample. One peak corresponded to real human reads, where mapped regions covered nearly the full read length, while the other peak, characterized by the low mapping length, spotted regions of accidental sequence similarity. We refer to the latter reads as false positives, as they incorrectly contributed to the observed fraction of human material in the samples.

To eliminate the noise coming from false positives, we increased the minimum seed length threshold for BWA to the 95th percentile of false positives’ mapping seed length (from 19 to 26 bases) (Fig. 5A,B). Increasing this threshold eliminated multimapping reads of low quality, now indicating two clear peaks corresponding to reads of a high probability of mapping correctly (MAPQ = 60) and those mapping to multiple regions equally well (MAPQ = 0) (Fig. 5C,D).

After increasing the threshold in BWA, we observed that for none of the BL samples, the human read content exceeded the expected 3% (Fig. 4A). For the KAPA and Nextera kits, the fraction of human reads in the samples slightly decreased with increasing homogenisation time, ranging from 2.83% for K1BL to 2.13% for K3BL and 2.88% for N1BL to 2.53% for N3BL, where 1/2/3 corresponds to 10/15/20 min of homogenisation time. For QIAseq, we observed a slightly higher fraction of human reads (2.95% for Q1BL to 3.11% for Q3BL), with Q2BL showing the lowest human read content of 2.6%.

More differences between isolation kits were observed in the results of mapping reads against the human genome from donor samples S1, S2 and S3 (Fig. 4B). Among all three kits, QIAseq gave the highest number of human reads (avg. 0.99%, sd. 0.61%) in comparison to KAPA (avg. 0.04%, sd. 0.03) and Nextera (avg. 0.1%, sd. 0.01). The impact of the library preparation kit was further supported by the results of PERMANOVA, in which we assessed the impact of the isolation kit and homogenisation time on the human reads fraction. The test showed that kits played a significant role in the fraction of reads mapped to the human genome (F value 74.012, R^2^ 0.866, p value 0.001), while neither homogenisation time (F value 0.916, R^2^ 0.012, p value 0.395) nor the combination of homogenisation time and kit (F value 0.787, R^2^ 0.018, p value 0.577) was significant (Table 2).

**Table 2:** Results of PERMANOVA test on the impact of homogenisation time and library prep kit on the number of human reads.

Independent variable	F-value	R^2^	p value
Homogenisation time	0.916	0.012	0.395
Library preparation kit	74.012	0.866	0.001
Homogenisation and kit combination	0.787	0.018	0.577
Residuals	–	0.105	–

**Figure 6:**
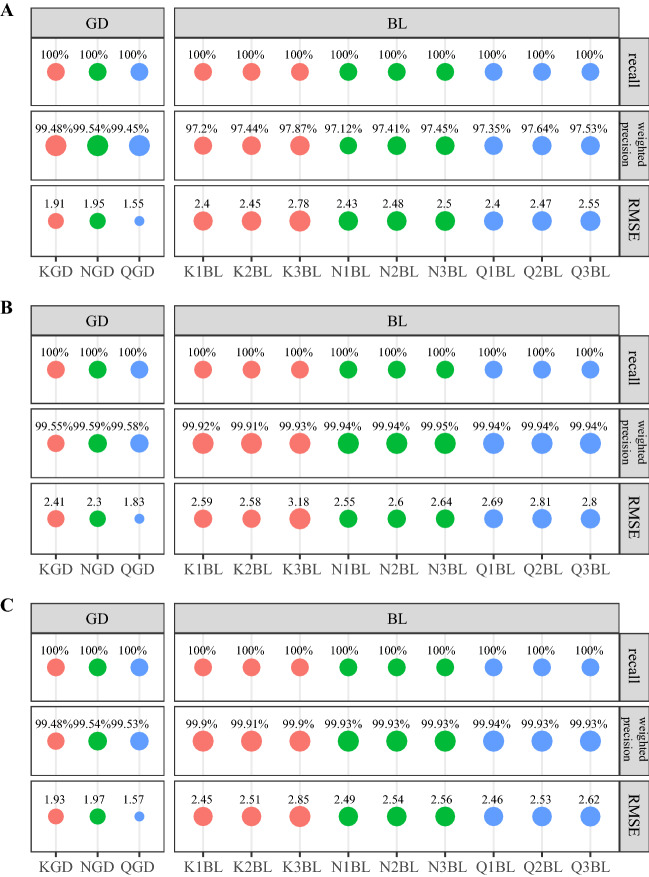
Bacterial community profiling metrics for the BL samples without filtering human reads (**A**) and with prefiltered human reads (BWA, seed length of 19 (**B**) and 26 (**C**)). Weighted precision metrics in (**A**) do not take into account reads classified as *Homo sapiens* species as TP (expected abundance of reads from *Homo sapiens* was estimated to be 2–3% of a sample). 1/2/3 corresponds to 10/15/20 min of homogenisation time.

We also checked how the prefiltering of human reads affects bacterial community profiling with Kraken2. We calculated recall, weighted precision and RMSE for GD and BL samples without filtering and with prefiltering of human reads with BWA with a seed length of 19 and 26 nucleotides (Fig. 6). Despite the fact that the results of mapping showed that BWA with a seed length of 19 gives a high amount of FP (reads classified as human species that in reality are of bacterial origin), our analysis revealed that prefiltering with lower (19) or higher (26) seed length does not affect the bacterial community profiling, giving comparable results to nonfiltered data. Although the weighted precision for unfiltered BL data was lower than that of filtered BL data (97.44% vs > 99.9%), it must be noted that weighted precision metrics for unfiltered data did not take into account reads classified as *Homo sapiens* species as TP. Moreover, as expected, the abundance of reads from *Homo sapiens* was estimated to be 2–3% of BL samples; the weighted precision for filtered and unfiltered data was comparable.

### Community reconstruction analysis for GD and BL samples

**Figure 7:**
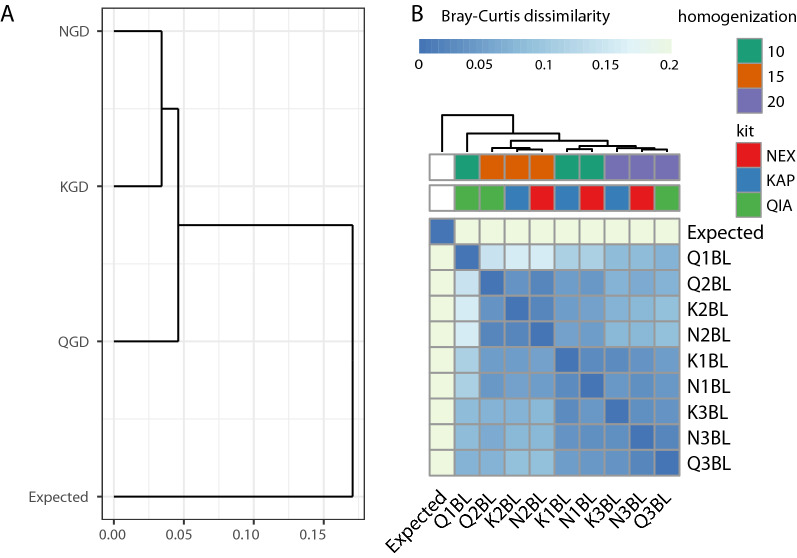
Bray–Curtis beta diversity clustering of GD (**A**) and BL (**B**) samples. Genomic DNA samples (GD) showed lower overall dissimilarity from the expected species composition than whole-cell isolates (BL). BL samples prepared with the same homogenisation times showed lower between-sample diversity. “Expected” refers to the known composition of samples specified by the manufacturer. 1/2/3 in the sample name corresponds to 10/15/20 min of homogenisation time.

The results of the community reconstruction analysis with Kraken2 for GD and BL samples can be found in Supplementary Table 8 (GD samples) and Supplementary Table 9 (BL samples). For both GD and BL samples, all of the expected species were identified by Kraken2—the recall parameter was equal to 100% for all the samples and tested conditions. The number of false discoveries was also low for both GD and BL samples and conditions (weighted precision exceeding 99.4% for all the samples and conditions), with slightly higher values obtained for BL samples (Fig. 6C, Supplementary Table 10). However, we did observe deviation from the expected species proportions, which was reflected in the RMSE of abundance, with the K3BL sample slightly deviating from the other samples (RMSE 2.85). Overall, GD showed a lower RMSE of correctly detected species abundance than BL (highest value 1.97 for NGD and lowest value 2.45 for K1BL). This was despite the fact that the GD sample composition was more taxonomically complex, with higher species diversity and staggered abundance. This observation was also supported by the similarity analysis. We observed a higher level of similarity to the original composition for GD than for BL; the highest Bray–Curtis dissimilarity value among GD was 0.17 (NGD, Fig. 7A, Supplementary Table 11), while the lowest dissimilarity of BL was 0.22 (K2BL, Fig. 7B). These observations point to the bias introduced by the isolation step in BL samples. The GDQ sample prepared with QIAseq was the most similar to the expected community, with a Bray–Curtis metric of 0.13 and RMSE of 1.57 (Fig. 7A, Supplementary Tables 12 and 13).

Clustering of the Bray–Curtis dissimilarity revealed that BL samples clustered by the homogenisation time rather than the library preparation kit (Fig. 7B, Supplementary Table 13). Samples homogenised for 20 min formed one cluster (average Bray–Curtis for K3BL/N3BL/Q3BL cluster was 0.079), and samples homogenised for 15 min formed a second cluster (average Bray–Curtis for K2BL/N2BL/Q2BL samples was 0.153). Although clustering revealed the Q1BL sample as a potential outlier, as it clustered separately from all the other samples due to a low beta-diversity, two other samples homogenised for 10 min, K1BL and N1BL, formed a solid cluster. Sample N1BL showed to be the closest to the expected composition among the BL samples, with beta-diversity of N1BL versus Expected of 0.219.

### Real samples profiling

**Figure 8:**
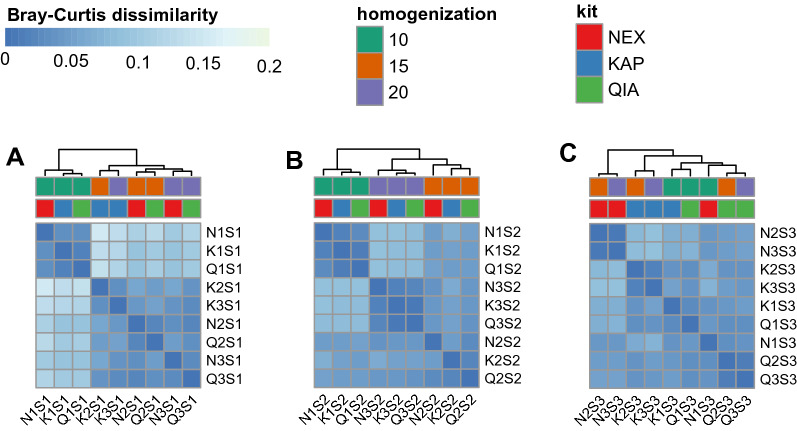
Bray–Curtis dissimilarity of donor samples S1 (**A**), S2 (**B**), and S3 (**C**) prepared with different kits and homogenisation times. Higher similarity was found between samples homogenised for 10 min than for 15- and 20-min homogenisation in S1 (**A**) and S2 (**B**) samples. For S3 (**C**), samples showed a low level of dissimilarity, with 10- and 15-min homogenisation clustering together within the same library preparation kit. 1/2/3 corresponds to 10/15/20 min of homogenisation time.

As opposed to the GD and BL mock samples, it was not possible to compare volunteers’ samples S1, S2, and S3 to any reference composition. To investigate the impact of isolation kits and homogenisation time on gut microbiome profiling, we calculated Bray–Curtis dissimilarity between samples coming from the same donor prepared with each of the protocols (Fig. 8, Supplementary Table 13)**.** We observed that for each of the S1 and S2 samples, 10 min of homogenisation resulted in more distinct communities than those observed between 15 and 20 min of homogenisation. This observation was less distinct for the S3 sample.

Moreover, while the S2 sample showed clear clusters of samples isolated with the same homogenisation time, for two other samples, we observed that after the homogenisation time was increased from 10 to 15 and 20 min, samples tended to cluster into small groups by the isolation kits. For S1, we observed a distinct cluster of KAPA samples (K2S1, K3S1) and a cluster of Nextera and QIAseq, further split by homogenisation time (N2S1 with Q2S1 and N3S1 with Q3S1). For S3, we observed distinct clusters of N2S3 + N3S3 and Q2S3 + Q3S3 and K2S3 + K3S3. Taxonomic profiles obtained for volunteer samples can be found in Supplementary Table 14, and the taxonomical tree for the Bacteria kingdom can be found in Supplementary Figs. 3, 4 and 5 for samples S1, S2 and S3, respectively. There are some differences visible within kits and homogenisation time for a given sample (Supplementary Figs. 6, 7, 8, 9, 10 and 11). Results of pairwise comparisons between different kits and different homogenisation times for each sample (S1/S2/S3) for Bacteria kingdom clades can be found in Supplementary Table 15.

### Gram-positive and gram-negative species isolation in BL samples

**Figure 9:**
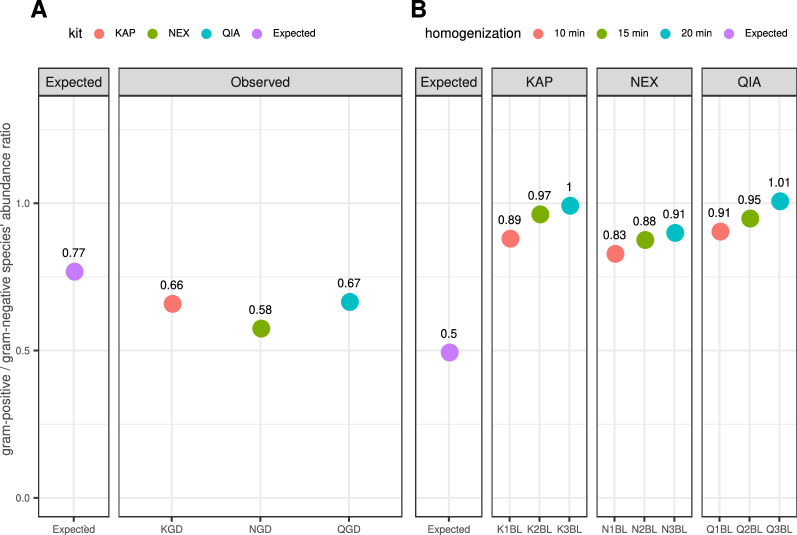
Expected versus observed proportion of gram-positive to gram-negative species in control sample GD (**A**) and isolated BL samples (**B**). For BL, we observed a higher fraction of gram-positive species than in the original bacterial mix, and the proportion of gram-positive bacteria increased with longer homogenisation times.

We assessed gram-positive/gram-negative species isolation bias in BL samples, as well as in volunteers’ samples. Additionally, we checked the gram-positive/gram-negative species’ abundance ratio in the GD samples that originated from a genomic DNA mix (Fig. 9A). Our analysis showed that overall, the proportion of gram-positive to gram-negative species’ abundance in GD samples was lower than expected. Since those samples were not subjected to isolation, we attribute the general differences to the difference in the genomes’ length (average genome length 2,536,718 bp for gram-positive and 4,282,149 bp for gram-negative species), which may have resulted in proportionally less gram-positive species’ genomes captured by the reads. Since gram-positive species had lower GC content than gram-negative species’ genomes (average 36.24% and 49.57%, respectively), we ruled out an amplification step as a potential source of differences in the species’ abundance. Moreover, we observed the Nextera kit to give results slightly more distant from the expected than KAPA and Qiagen kits. The largest differences in abundance between KGD and NGD were present for *Escherichia coli* (gram-negative species, 0.183% in KGD vs 0.211% in NGD) and *Staphylococcus epidermidis* (gram-positive species, 0.187% in KGD vs 0.160% in NGD). For QGD and NGD, the largest observed difference in abundance was for *Rhodobacter sphaeroides* (gram-negative species, 0.265% and 0.231% abundance, respectively).

**Figure 10:**
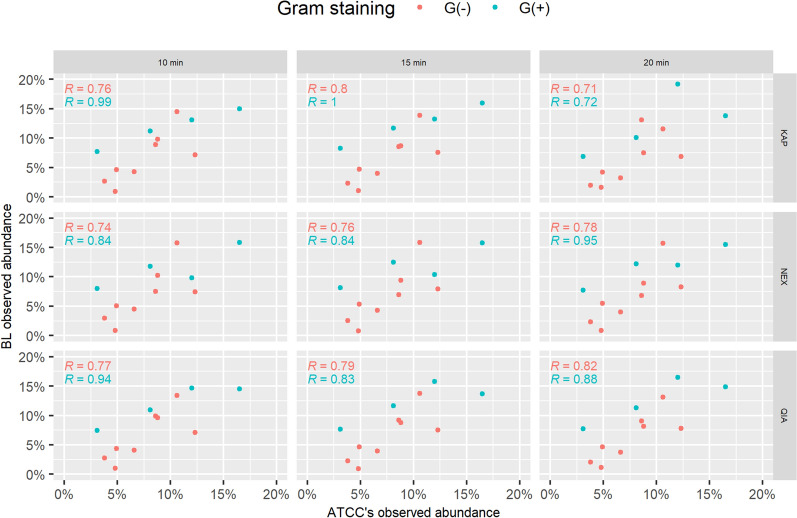
Species abundance observed for ATCC^®^ MSA-2006™ versus our observed abundances, labelled by Gram stain status. For both gram-positive and gram-negative species, our results correlate with the producer’s findings; however, for gram-positive species, the correlation was not significant in all the samples, as there were only 4 g-positive species in ATCC^®^ MSA-2006™.

In the BL bacterial mix with an even species abundance distribution, we expected to see twice as few gram-positive species as gram-negative species (gram-positive/gram-negative ratio 0.5). However, for all the kits, as opposed to the GD samples, we observed a higher fraction of gram-positive species than expected (avg. gram-positive/gram-negative ratio 0.885), which consistently increased with homogenisation time (Fig. 9B). We further compared the observed abundance of both gram-positive and gram-negative species with abundances reported by ATCC producer^38^ (Supplementary Table 1) for ATCC^®^ MSA-2006™ to investigate whether the bias in species abundance that we observed was consistent with those results, which resulted in a gram-positive/gram-negative species ratio of 0.657. Nevertheless, for both gram-positive and gram-negative species, we observed a linear correlation of abundances, indicating a positive relationship between our abundance and the producer’s abundance (Fig. 10). Correlation coefficients for gram-negative species ranged between 0.71 in K3BL and 0.82 in Q3BL, while correlation coefficients for gram-positive species ranged between 0.72 in K3BL and 1 in K2BL.

While zooming in to the deviations from the expected abundance, which was 8.3% for all the species in the BL sample, we observed groups of species that were systematically either over- or underrepresented (Supplementary Fig. 12). Among species with higher abundance than expected, we identified *Bifidobacterium adolescentis* (median abundance 13.24%), *Clostridioides difficile* (median abundance 14.97%) and *Lactobacillus plantarum* (median abundance 11.62%). Underrepresented species included *Escherichia coli* (median abundance 4.04%), *Helicobacter pylori* (median abundance 2.33%), *Fusobacterium nucleatum* (0.96%) and *Salmonella enterica* (4.66%). While 2 out of 3 overrepresented species belonged to the gram-positive group of bacteria (namely *B. adolescentis* and *C. difficile*), all the underrepresented species belonged to the gram-negative group.

We investigated the possible impact of Gram staining status, genome length and GC content on the median abundance of the species across BL samples. We fit a linear model with a top-down approach, which showed that in our data, only the Gram staining status is a significant predictor of median abundance, with the coefficient of 0.0634 and a *p *value of 0.0156 for the final model, which included Gram staining status only (Supplementary File 2).

### Gram-positive and gram-negative species isolation efficiency

We observed mapped reads for Zymo-spikes, not only in spiked volunteer samples S1, S2 and S3, where the Zymo control was added but also in BL and GD samples without spikes (Supplementary Table 16). However, the overall fraction of these Zymo-spikes’ genomes covered was much lower for BL and GD samples (1.08–1.81% for *Allobacillus halotolerans* and 0.31–0.42% for *Imtechella halotolerans*) than for volunteer samples (5.65–43.06% for *Allobacillus halotolerans* and 7.36–73.06% for *Imtechella halotolerans*). Additionally, the median coverage of those regions was much higher in BL and GD (ranging between 21 × and 207 × for *Allobacillus halotolerans* and 17 × to 227 × for *Imtechella halotolerans*) than in volunteer samples (median coverage of 1 × for *Allobacillus halotolerans* and 1–2 × for *Imtechella halotolerans*). This suggests that reads mapping to Zymo-spikes in BL and GD originate from a narrow range of highly similar regions across species, while in volunteer samples, there is low but consistent coverage along the expected genomes from spikes. Nevertheless, regions similar across the whole microbial community would impact mappings in the volunteer samples; therefore, inferring gram-positive/gram-negative species isolation bias required analysis of reads’ coverage rather than directly comparing a fraction of mapped reads.

Having a median coverage of 1 × across most of the genomic sequence for the volunteer samples, the proxy for strain abundance was a fraction of the genome that was observed in sequencing reads. The ratio of the genome positions that were covered by reads during mapping to the reference genomes of *Allobacillus halotolerans and Imtechella halotolerans* suggests that in our experiment, a slight bias toward more efficient isolation of gram-negative species could be observed for real samples (25.47% of the *Allobacillus halotolerans* genome covered compared to 42.50% for *Imtechella halotolerans*). This observation was supported by the Wilcoxon test, resulting in a p value of 1.9e−04. However, in volunteer samples, we did not observe a significant impact of homogenisation time, kit or combination of both on the *Allobacillus halotolerans/Imtechella halotolerans* ratio, as supported by PERMANOVA (Table 3).

**Table 3:** Results of PERMANOVA test on impact of homogenisation time and library prep kit on gram-positive/gram-negative species isolation bias (*Allobacillus halotolerans/Imtechella halotolerans* ratio).

Independent variable	F value	R^2^	p value
Homogenisation time	0.433	0.045	0.642
Kit	0.180	0.019	0.813
Homogenisation time × kit	0.019	0.004	1.000
Residuals	–	0.933	–

## Discussion

The gut microbiome is being actively studied by many research groups. Although much effort has been put into understanding how various metagenomic analyses may influence the obtained results, great variability in approach, methodology, result representation and interpretation are still routinely reported. These inconsistencies may be a source of a significant misinterpretation of data and ambiguous results. In our study, we investigated the procedure of DNA isolation, preparation of NGS libraries and bioinformatics analysis, allowing for the identification of microbiome taxa in the collected samples. We reported that choices made at each step of the procedure influenced the results, with a more substantial bias introduced by different NGS library kits and tools aimed at identifying microbial taxa.

### Impact of sampling/storage stages on results

Preservation of the microbial DNA that is contained in the stool sample is crucial for the precise identification of the microbiota. Many publications examine the impact of sampling and storage procedures on the final results of metagenomic analysis. In an ideal case, the collected samples should be transported to the laboratory as soon as possible, and DNA should be isolated immediately after delivery. It is acceptable for the stool sample to be kept without preservation for a maximum of 4 h^13,39,40^. However, this scenario is not always possible, as the study participants need time to deliver the sample, and the transportation time is usually much longer than 4 h. Meanwhile, too high of a temperature may cause undesirable bacterial growth that will significantly influence the analysis of the sample’s content. Similarly, preserving aerobic conditions resembling the gut microenvironment may also be crucial for maintaining the original microbiota in stool samples. Studies show that the sample could be stored at ~ 4 °C for 24 h to 48 h without significant microbial composition alteration^41–45^. For longer storage, − 20 to − 80 °C is required^38,46,47^. However, various authors emphasized that the temperature during transport should be controlled, as it is known that frozen-thaw cycles and repeated heating processes can cause serious bacterial DNA damage and degradation, which bias the metagenomic analysis^41,42,48–50^.

Under real conditions, immediate transfer to the laboratory and freezing at − 80 °C, the temperature of transportation control, and storage at 4 °C for 24–48 h are almost impossible to implement. Therefore, although immediate freezing of the samples is referred to as the gold standard, various DNA and RNA preservation agents designed to prevent bacterial lysis and endonuclease activity are also often utilised. Using such preservation reagents allows the transportation time to be significantly extended without the need to store samples at a low temperature. One of the most well-known stabilisation buffers, RNAlater^®^, has been proven to protect DNA from degradation at room temperature for days to weeks^41,46,50–53^. Even though there is a concern that usage of the stabilisation buffer may bias the initial microbiome composition in the stool by the buffer itself^48,52,54^, Wu et al. reported only a mild deviation of microbial composition compared to the same faecal sample that was frozen immediately^55^. However, another study showed that for 16S rRNA gene sequencing, utilisation of a preservative agent such as RNAlater^®^ might be unnecessary for samples stored for up to 7 days at − 80 to 32 °C^56^.

Considering the pros and cons of various DNA preservation methods, in our research, we decided to minimize the sample delivery time (24–48 h) with the simultaneous use of the RNAlater^®^ stabiliser. This approach offers the right balance between the security of the stabilisation buffer and the efficiency of fast delivery times. After collection, our samples were homogenised, which is in line with the current recommendations. Due to intrasample variation, a sample should be homogenised to minimize the differences within the sample^41,46,50–53^. Homogenised samples were kept in a − 20 °C freezer until DNA extraction. Studies have reported that this temperature is able to maintain a stable microbial community for at least a few months^46,49^. However, if a more extended storage time is desired, a lower temperature of − 80 °C should be applied that is able to maintain the microbiota for up to 2 years^47^.

### Isolation biases

For DNA extraction from stool samples, we used the DNeasy PowerSoil Pro Kit (Qiagen, Germany). Our procedure resembles the one applied in the Human Microbiome Project. Mechanical disruption of microbial cell walls was indicated by the International Human Microbiome Standard (IHMS) as the step most affecting DNA isolation, especially for gram-positive bacteria and fungi^17^. This task is accomplished by the bead-beating of a sample. In our procedure, we tested this step three times: for 10, 15 and 20 min. We did not observe a significant impact of the homogenisation time on the amount of isolated DNA (Supplementary Table 4). The differences in the extracted DNA amount may be due to the nonhomogeneous nature of the stool sample material.

Moreover, we observed that gram-positive species were more abundant than expected for samples originating from the mock community ATCC^®^ MSA-2006™ (BL samples), with the gram-positive/gram-negative ratio increasing with homogenisation time (Fig. 9). We compared our results with abundances that the producer has reported in shotgun sequencing, and while the gram-positive/gram-negative ratio was lower than that in our experiment, the species abundances we identified correlated with those reported by ATCC (Fig. 10).

At the same time, we observed a significant difference between gram-positive and gram-negative species from Zymo-spikes (*Allobacillus halotolerans*/*Imtechella halotolerans* ratio, Wilcoxon test, *p *value of 1.9e−04, Table 3) toward a higher mean fraction of the covered genome of gram-negative *I. halotolerans*. Although we found reads also mapping to those genomes in the samples which were not spiked, we showed that such false positive identifications are represented by short regions of high coverage (possibly originating from regions common across species), while for the true positive detection of *I. halotolerans* and *A. halotolerans* in spiked samples the coverage was low, but spanned across a broader range of the genomes’ positions. The difference in coverage in spiked samples, however, was not related to the isolation kit or homogenisation time used, as confirmed by PERMANOVA on the impact of homogenisation time and library prep kit on the *Allobacillus halotolerans/Imtechella halotolerans* ratio. Two additional factors may come into play for *A. halotolerans*/*I. halotolerans* ratio, which are differences in the genome lengths (2700 Mbp for *A. halotolerans* vs 3113 Mbp for *I. halotolerans*) and GC contents of the genomes (39.7% for *A. halotolerans* and 35.6% for *I. halotolerans*). Although subtle, those differences, when considered in addition to the gram-positive status of *A. halotolerans*, may be adding up to the lower coverage of this species. As for the potentially multi mapping reads, the behaviour of the BWA algorithm used is such that if the read maps equally well to more than one region of the reference genome, the read is assigned randomly to one of them with a low mapping quality score. Therefore, we do not expect multi mapping reads to inflate the coverage.

Our observations of isolation bias toward gram-negative species for Zymo-spiked human samples are in line with previous reports indicating that lysis of the cellular wall of gram-positive bacteria may be an obstacle for isolation kits, resulting in a lower amount of gram-positive DNA material in prepared samples^13,57,57–63^. However, results obtained for BL samples are contradictory as we observed bias towards lower abundance of gram-negative species rather than gram-positive. For ATCC^®^ MSA-2006™, which is a much less complex community composed of 12 strains in equal abundance, homogenisation times as high as 10, 15 and 20 min might have led to mechanical shearing of more fragile gram-negative species DNA^64^, reflected in lower species abundance in the Kraken2/Bracken pipeline. In this case, we ruled out a potential impact of genome lengths and GC content on the abundance estimates with a linear model, which confirmed the impact of Gram staining status on the median species’ abundance. We also identified specific species which were under- and over-represented in the reconstructed mock community, and those results showed to be in line with the gram-status significance, with all the under-represented species belonging to the gram-negative group.

Altogether, results obtained from BL and Zymo-spiked human samples show that several factors may come into play when considering the impact of homogenisation on the isolation biases of gram-positive and gram-negative bacteria. While the lysis of gram-positive bacteria cellular wall may be an obstacle for DNA isolation, the fragileness of gram-negative bacteria may cause mechanical fragmentation of their DNA. Depending on which phenomena prevail, we can observe either under-represented gram-positive or gram-negative species. Our results suggest that, 10 min of homogenisation best balances these two opposing factors, allowing a better reflection of gram-positive and gram-negative bacteria.

Moreover, while the result of the PERMANOVA test on the impact of homogenisation time and library prep kit on the number of human reads (Table 2) showed the impact of the library preparation kit rather than the homogenisation time on the human DNA content, analysis of both: mock samples BL and human faecal samples revealed that the time of homogenisation has an impact on the beta-diversity of the communities. Samples homogenised for 10 min were distinct from samples homogenised for 15 and 20 min (Figs. 7B, 8). In general, these samples formed also a more solid cluster.

These results show that the homogenisation time is an essential factor that may influence the profiling of microbial communities. Ten minutes of homogenisation seems a favourable time, taking into account the consistency of the obtained results and the trade-off between the difficulty in lysis of gram-positive bacteria and the possibility of damaging the gram-negative bacteria DNA. These results also suggest possible DNA degradation of the shorter genomes with the increase of the homogenisation time. Additionally, it is worth mentioning that one of the previous works concerning the impact of the DNA extraction from faecal material on the microbial community structure reported significant differences between the two investigated methods despite comparable bead-beating steps applied in the two investigated methods^13^. In this case, the amount of DNA was highly dependable on the procedure applied, resulting in further differences at the taxonomy levels.

### Impact of kits

Many efforts have been made to study the impact of NGS library kits on the obtained results^65–67^. The overall conclusion is that the choice of a library preparation kit can strongly affect the results. All NGS library preparation kits assessed in this study were PCR-based. The main difference between kits is that the Nextera kit utilises a tagmentation method to generate DNA fragments, while Qiagen and KAPA utilise enzymatic fragmentation. It must be noted that several studies highlighted the advantage of PCR-free-based approaches over PCR-based approaches. Jones et al. showed that the usage of PCR-free-based approaches can reduce bias in the calculation of abundance and improve assemblies for the accurate taxonomic assignment^65^. In Jones’ study, PCR-free methods generated much longer contigs, had much lower duplication rates, and low numbers of low-quality reads compared to PCR-based methods. Another study showed that PCR amplification during library preparation can introduce some bias in low-GC regions^67^. However, in metagenomic studies, the extracted amount of DNA can be too low to use PCR-free methods, which require 1–2 µg of DNA (according to the manufacturer’s guidance). Moreover, PCR implementation improves the quality of the library and guarantees that more DNA fragments will contain ligated adapters and generate clusters on a sequencing flow cell. In our approach, we applied only 6 PCR cycles, which seems to be a reasonable compromise between PCR-free and PCR-based methods.

Among the libraries that we tested in this study, Nextera gave the best results under most of the assessed criteria. It reflected the most homogeneous and repeatable results in terms of library preparation and diversity of the reconstructed mock communities. This may be due to the utilisation of the tagmentation method, as fragmentation based on the Tn5 transposase has been described as a highly efficient DNA fragmentation method^68^.

In our study, the Nextera kit produced the least variable number of reads, regardless of the homogenisation time (Fig. 2A, Supplementary Table 6), which can be considered a pro in terms of reproducibility and further *k*-mer-based community reconstruction that is used by Kraken2. Moreover, the content of human material in donor samples was very low in samples prepared using the Nextera NGS library kits (Fig. 4B). However, this phenomenon cannot be easily explained, and the KAPA kit also showed good results in terms of the human material content. Of note, our experience shows that by manoeuvring the DNA isolation parameters, it is possible to increase the abundance of bacterial DNA in the isolate^69^ (e.g. by longer incubation at a higher temperature, which results in better DNA recovery from gram-positive bacteria with a thick cell wall). Indeed, detailed protocols for microbial DNA isolation advise additional incubation at high temperature for increasing DNA yield from gram-positive bacteria. Faecal samples contain a complex array of polysaccharides, lipids, salts and cells. Heating the sample increases the reaction rate between the lysis buffer (Solution CD1 from the kit used contains SDS and other disruption agents that aid cell lysis. SDS is an anionic detergent that breaks down fatty acids and lipids associated with the cell membrane of several organisms.) and these substances, and as a result, aids cell lysis.

Additional evidence supporting the importance of the choice of the kit during profiling metagenomic communities comes from the analysis of genomic DNA samples—NGD sample prepared with Nextera showed a slightly lower gram-positive/gram-negative species’ abundance ratio than samples prepared with KAPA (KGD) and Qiagen (QGD) (Fig. 9A). At the same time, the two latter kits showed the same ratio, which may be attributed to the differences in the biochemistry underlying the library preparation between Nextera and KAPA/Qiagen. Nevertheless, all the samples showed a lower ratio of gram-positive species than expected from the original sample composition, which highlights possible overall biases introduced by the library preparation in line with the genomes’ lengths. However, more definite conclusions would require further investigation on more replicates.

Statistical analysis of the human content in the BL samples also revealed that the choice of library prep kit plays a more significant role than homogenisation times in terms of the number of human reads, as confirmed by the results of the PERMANOVA test (*F *value = − 74.01, *p *value = 0.001, Table 2). However, the higher complexity of real stool samples might underlie the significance of homogenisation time, with 10 min of homogenisation being clearly distinct from 15 and 20 min homogenisation (Fig. 8).

### Impacts of software and parameters

One of the most critical steps in the whole metagenomic analysis is the correct assignment of obtained reads to the taxa to which they belong. In our study, we tested two leading methods for metagenomics taxa identification: MetaPhlAn2 and Kraken2/Bracken combination. In our experiment, the Kraken2/Bracken combination proved to outperform MetaPhlAn2 in terms of recall, and while it resulted in more false positive species being identified, the fraction of those in the overall sample composition and the abundance error of the correctly identified species remained low maintaining good weighted precision and RMSE (Fig. 3). This was true regardless of the tested Kraken2 confidence threshold values and type of in silico species distribution. This is in line with the results of the recent benchmark of metagenomics tools for taxonomic classification^70,71^. This might be caused by the fact that MetaPhlAn2 is based on marker genes, which might be absent when the sequencing depth is low.

According to this benchmark, Kraken2 was among the best-scoring methods, while in general, marker-based tools performed worse. Additionally, Bracken—a postprocessing step intended to improve abundance estimates by Kraken2—provided more accurate abundances at the species level. Kraken2 and its companion tool Bracken also provide good performance metrics and are very fast on large numbers of samples. One of the main drawbacks of Kraken2 is its large computational memory requirement, especially compared to tools such as MetaPhlAn2^72^. However, importantly, the current implementation of Kraken2 is much more efficient in terms of memory usage than the previous version of Kraken. Kraken2 reduces the memory usage by 85% compared to Kraken1, allowing greater amounts of reference genomic data to be used, while maintaining high accuracy and increasing speed five-fold^21^. But even if Kraken2 reduces the amount of memory needed and its actual demand depends on the dataset analysed, it still may need ~ 40 GB memory for an average metagenomic dataset^72^. In this context, MetaPhlAn2 is a good choice when only limited computational resources are available, as it has very low computational requirements (< 2 GB of memory) and fast classification speed. However, it must be noted that, contrary to Kraken2, MetaPhlAn2 does not allow for the use of custom databases, which may be a serious obstacle when performing metagenomic analyses. The application of the lightweight Kraken Mini database may also be a solution for low-performance hardware.

An additional aspect that could bias the final taxonomy profiling results is mapping against the reference genome of a human or potential contaminant that is not present in the databases of tools for taxonomy profiling. As we previously mentioned, this step, if not performed mindfully, can lead to a high fraction of reads that map only with a short overlap and result more likely from the regions common across species (Fig. 5). This can further result in removing bacterial reads that share sequence similarities with the reference genome, leading to a less accurate bacterial community reconstruction (Fig. 6). Increasing the minimum seed length threshold proved to be a good solution in this situation, yielding more accurate alignments (Fig. 4). On the other hand, Kraken2 proved to deal very well with human reads, even if they were not filtered out, and no significant differences for either GD or BL sample metrics were observed, regardless of whether human reads were filtered out (Fig. 6). It is likely that reads that falsely mapped to the human genome with the seed of 19 nt have matched to non-specific regions and would not be discriminative on the microbial species level in Kraken2 profiling. Therefore, while controlling the amount of human material is recommended, the step of filtering out reads originating from human material or contaminants does not necessarily have to be introduced in the bioinformatics pipeline prior to taxonomy assignment when the Kraken2/Bracken tool is utilised. Our experiment showed that it does not necessarily lead to a loss of significant data and does not negatively impact the reconstruction of the community. This finding is especially valuable considering that mapping reads to the human genome is usually time-consuming, and using tools such as Kraken2, even with the whole set of raw metagenomic reads, is much faster than making full alignment to the human genome.

Another source of potential problems can be a bias introduced by the regions of high similarity shared by different bacterial species. This may lead to a situation in which some reads are inappropriately classified, giving false positives and false negatives. Although it is not possible to completely eliminate this problem, tweaking parameters for taxonomic reconstruction may improve the results, as shown by the results of the in silico profiling (Fig. 3). More importantly, in silico analysis of samples mimicking ATCC^®^ MSA-2006™ composition with even and staggered abundance, as well as analysis of GD and BL samples, showed that using an appropriate tool with tweaked parameters minimizes the risk of false negatives (recall metric of 100%) and, at the same time, the differences in real and detected species abundance are low, as revealed by the weighted precision and RMSE metrics (Figs. 3, 6A). Moreover, the lower RMSE for the in silico staggered sample (ISS) shows a weaker impact of abundance error for low-abundance species.

Selecting a tool for microbiome profiling is one of the key aspects of performing metagenomic analyzes. Unfortunately, there is no single, simple answer to which tool is better. Depending on the specificity of the analysed data and the metrics considered, various tools may perform better or worse in individual benchmarks. For example, one of the conclusions drawn from the CAMI2 competition^72^ is that methods based on gene markers work well in profiling the intestinal microbiota. Contrary to that, our research indicates the advantage of the Kraken2/Bracken combination (higher recall, lower abundance error of the correctly identified species, higher weighted precision) over MetaPhlAn2. Methods based on *k*-mers, like Kraken2, were also among the best-scoring in the Ye et al. benchmark^70^.

For analyses of the taxa composition, tools based on genome fragments may be sufficient. But for the reconstruction of whole genomes present in a sample, an approach based on the whole genomes is needed. In such a case, the crucial aspects are depth of sequencing and proper preprocessing of the data^72^. Another aspect to consider is the reference database. It is essential that the program has a sufficiently large and up-to-date database of reference genomes or allows the users to create a custom database. The latter is not possible for all tools; for example, already mentioned MetaPhlAn2 does not allow a custom database creation. This aspect is also addressed as essential to consider by Ye et al. in their benchmark^70^. The choice of a taxonomic profiling abundance tool may be also dictated by practical aspects such as the availability of computing resources or the operating time.

Considering the lack of a golden standard regarding the choice of a metagenomic profiling tool, in order to choose the right tool for metagenomic analyses, it is important to focus on the purpose of the analysis and the specificity of the data (type of data, depth of sequencing, etc.). It is most desirable when the specificity of the data and the method of their preparation (isolation, NGS libraries’ construction, selection of the platform and sequencing parameters) are appropriately selected in terms of the research objective.

## Conclusions

The design of an experiment and the detailed establishment of an experimental protocol may have a serious impact on determining the taxonomic profile and community content of the intestinal microbiome. During the experiment, we observed that bacterial taxonomic profiles may be biased. This is mainly due to the details of the library preparation methods, as well as different bioinformatics tools. Therefore, there is an urgent need to standardise the procedure of intestinal microbiota determination. To understand and reduce the bias introduced during experiments, we carefully analysed the consecutive steps of the procedure that allowed for the detection of microbiome composition. As a result, we present recommendations that allow for the optimisation of metagenomic analysis of the intestinal microbiota.

We have shown that while the time of homogenisation does not seem to impact gram-positive/gram-negative isolation efficiency, it may affect their composition. Our findings suggest that the homogenisation time is the leading factor impacting sample diversity. We would recommend 10 min of homogenisation as it allows to better reflect the gram-positive/gram-negative ratio, and the obtained results are the least diversified in terms of beta-diversity. Of notice, our analysis revealed that the choice of the library preparation kit influences the repeatability of the results, which is an important factor that has to be taken into account, especially in metagenomic experiments, where a high variability is observed. In this study, the Nextera kit, which is based on a tagmentation method, allowed us to obtain the most reproducible results. Moreover, the choice of computational tools and their parameters are crucial for reliably determining the content of intestinal microbiota, as proven by the Kraken2/Bracken pipeline outperforming MetaPhlAn2 in our experiments in terms of high recall and a low contribution of falsely identified species in the final sample composition. We believe that our findings can be helpful for a wide range of subsequent studies that aim to better understand the role of the gut microbiome, as well as for clinical purposes, where the optimisation of the metagenomic pipeline and understanding of its influence on the final results may have a direct impact on the diagnosis.

## Supplementary Information

Supplementary Information 1.

Supplementary Information 2.

## Data Availability

The raw datasets generated and analysed during the study are available in the NCBI SRA repository [PRJNA749919].

## Abbreviations

BL (ATCC bacterial mix): Gut microbiome whole cell mix (ATCC^®^ MSA-2006™)
FP: False positive species
GD (ATCC genomic DNA): Gut microbiome genomic mix (ATCC^®^ MSA-1003™)
ISE: In silico generated samples with even abundance
ISS: In silico generated samples with staggered abundance
KAPA: KAPA HyperPlus (Roche, Switzerland)
Nextera: Nextera DNA Flex Library Prep (Illumina, USA)
NGS: Next generation sequencing
PBMC: Peripheral blood mononuclear cells
RMSE: Root mean square error
Qiagen: QIAseq FX DNA Library Kit (Qiagen, Germany)
S1, S2, S3: Real human faecal samples analysed during the study
TP: True positive species
