# Supplementary material for: The standardisation of the approach to metagenomic human gut analysis: from sample collection to microbiome profiling
Source: Sci Rep. 2022 May 19;12:8470. doi: 10.1038/s41598-022-12037-3 (PMC9120454; doi:10.1038/s41598-022-12037-3)
Supplement: Supplementary file 1 — Supplementary Information 1. [file 41598_2022_12037_MOESM1_ESM.docx]

# Supplementary materials

**Supplementary Figure 1.** **The number of papers on the microbiome from 1980 to 2021 stored in the PubMed database**. (“Microbiome” or “microbiota”) was used as a search condition.

**Supplementary Figure 2. Agarose-gel electrophoresis of the microbial DNA extracted from stool samples of three donors (S1, S2, S3) with Zymo-spikes, using three different homogenisation times (10, 15 and 20 minutes)**. The 500 ng of DNA per extraction was loaded and electrophoresed on a 1% agarose gel, stained with Midori Green (Genetics), and DNA ladder (Ready-to-Use DNA Size and Mass Standard from DyNAzyme EXT PCR Kit (Finnzymes) in the first lane was used as a ladder [Control].

**Supplementary Figure 3. Taxonomic trees for S1 samples (Bacteria kingdom).** The size of nodes is set according to the number of different clades at a given taxonomical level. Colour is intensified according to the number of reads for a given clade.

**Supplementary Figure 4. Taxonomic trees for S2 samples (Bacteria kingdom).** The size of nodes is set according to the number of different clades at a given taxonomical level. Colour is intensified according to the number of reads for a given clade.

**Supplementary Figure 5. Taxonomic trees for S3 samples (Bacteria kingdom).** The size of nodes is set according to the number of different clades at a given taxonomical level. Colour is intensified according to the number of reads for a given clade.

**Supplementary Figure 6. Graphical representation of the pairwise comparisons between different kits for the S1 sample for Bacteria kingdom clades.** The size of nodes on tree figures is set according to the number of different clades at a given taxonomical level, while the colour is intensified according to the log foldchange value for a given clade.

**Supplementary Figure 7. Graphical representation of the pairwise comparisons between different homogenisation times for the S1 sample for Bacteria kingdom clades.** The size of nodes on tree figures is set according to the number of different clades at a given taxonomical level, while the colour is intensified according to the log foldchange value for a given clade.

**Supplementary Figure 8. Graphical representation of the pairwise comparisons between different kits for the S2 sample for Bacteria kingdom clades.** The size of nodes on tree figures is set according to the number of different clades at a given taxonomical level, while the colour is intensified according to the log foldchange value for a given clade.

**Supplementary Figure 9. Graphical representation of the pairwise comparisons between different homogenisation times for the S2 sample for Bacteria kingdom clades.** The size of nodes on tree figures is set according to the number of different clades at a given taxonomical level, while the colour is intensified according to the log foldchange value for a given clade.

**Supplementary Figure 10. Graphical representation of the pairwise comparisons between different kits for the S3 sample for Bacteria kingdom clades.** The size of nodes on tree figures is set according to the number of different clades at a given taxonomical level, while the colour is intensified according to the log foldchange value for a given clade.

**Supplementary Figure 11. Graphical representation of the pairwise comparisons between different homogenisation times for the S3 sample for Bacteria kingdom clades.** The size of nodes on tree figures is set according to the number of different clades at a given taxonomical level, while the colour is intensified according to the log foldchange value for a given clade.

**Supplementary Figure 12. Graphical representation of the deviations from the expected abundance for the species in BL samples for all three investigated times of homogenisation (10, 15 and 20 minutes).** The dotted line represents the expected ratio for species in BL samples.

**Supplementary Table 1. Strain characteristics of bacteria from ATCC bacterial mix, ATCC genomic DNA, and ZymoBIOMICS™ Spike-in Control I**

**Supplementary Table 2. *In silico* generated sample parameters**

**Supplementary Table 3. Species abundance in simulated NGS samples ISE and ISS**

**Supplementary Table 4. Quality and quantity of isolated DNA measured by a Nanodrop ND-1000 spectrophotometer (Thermo Fisher Scientific, USA)**

**Supplementary Table 5. Protocol comparison of library preparation kits used in the study**

**Supplementary Table 6. Statistics for reads before and after preprocessing**

**Supplementary Table 7. Top undetermined barcodes**

**Supplementary Table 8. Community reconstruction metrics on *in silico* samples obtained with MetaPhlAn2 and Kraken2/Bracken with default parameters and increased confidence threshold for Kraken2**

**Supplementary Table 9. Community profiles of *in silico* samples ISE and ISS obtained using MetaPhlAn2 and Kraken2/Bracken with various Kraken2 confidence thresholds**

**Supplementary Table 10. The community reconstruction of the GD samples obtained using Kraken2/Bracken with the 0.1 threshold**

**Supplementary Table 11. The community reconstruction of the BL samples obtained using Kraken2/Bracken with the 0.1 threshold**

**Supplementary Table 12**. **Community reconstruction merics for GD and BL samples**

**Supplementary Table 13. Bray-Curtis dissimilarity for GD, BL and volunteers’ samples**

**Supplementary Table 14.** **Taxonomic profiles of volunteer samples prepared with KAPA, Nextera and QIASeq kits with 10, 15 and 20 minutes of homogenisation**

**Supplementary Table 15.** **Results of pairwise comparisons between different kits and different homogenisation times for each sample (S1/S2/S3) for Bacteria kingdom clades.** Pairwise comparisons were performed with the Wilcoxon Rank Sum test on the differences in median abundance for each tested condition (kits/homogenisation) for each sample (compare_groups function, metacoder R package). The results were further adjusted for multiple comparisons with the FDR method (mutate_obs function, metacoder R package and p.adjust function, stats R package) – wilcox_p_value.

**Supplementary Table 16. The fraction of the reference genomes covered with at least one read and the median coverage of covered regions. For BL and GD samples, where zymospikes were not added, false positive mappings to genome regions shared between multiple species were observed.**

**Supplementary File 1. Quality statistics for DNA library preparation with KAPA, Nextera, and Qiagen**

**Supplementary File 2.** **Results of the linear regression investigating the possible impact of Gram staining status, genome length and GC content on the median species abundance across BL samples.** The initial model included all three mentioned independent variables, with the median abundance as a dependent variable with *lm* function in R (stats R package). The least significant variable was iteratively removed from the model until a final model including only variables significant for the median abundance was reached.
