# Supplementary material for: The standardisation of the approach to metagenomic human gut analysis: from sample collection to microbiome profiling
Source: Sci Rep. 2022 May 19;12:8470. doi: 10.1038/s41598-022-12037-3 (PMC9120454; doi:10.1038/s41598-022-12037-3)

Quality statistics for DNA library preparation with KAPA

SAMPLE	LIBRARY ID	QUBIT		AGILENT
		ng/ML	ng/ul	Average size (bp)
K1BL	KAPA	500	100	719
K2BL	KAPA	335	67	784
K3BL	KAPA	25,1	5,02	1862
K1S1	KAPA	77	15,4	1196
K2S1	KAPA	27,7	5,54	1574
K3S1	KAPA	30,7	6,14	1599
K1S2	KAPA	590	118	738
K2S2	KAPA	116	23,2	771
K3S2	KAPA	493	98,6	635
K1S3	KAPA	461	92,2	1183
K2S3	KAPA	37,4	7,48	1382
K3S3	KAPA	29,9	5,98	1350
KGD	KAPA	24,2	4,84	961

K1BL

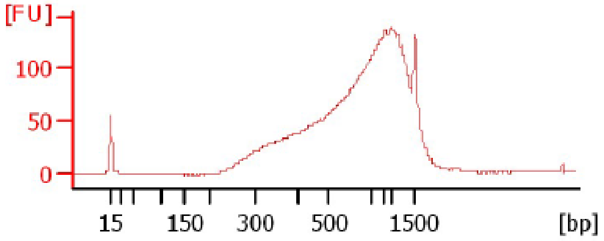

K2BL

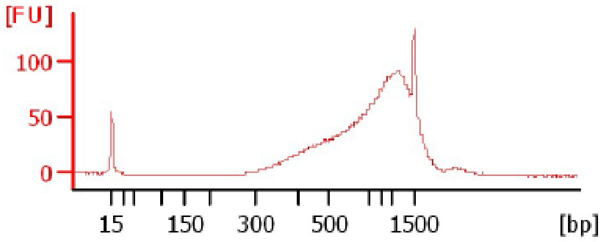

K3BL

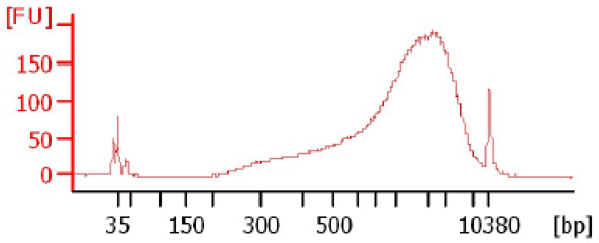

K1S1

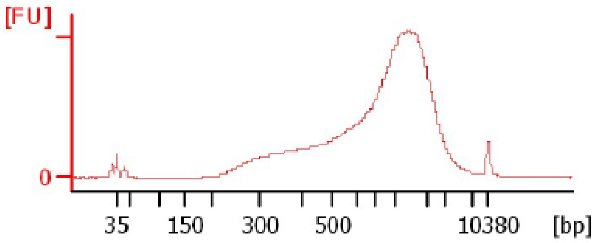

K2S1

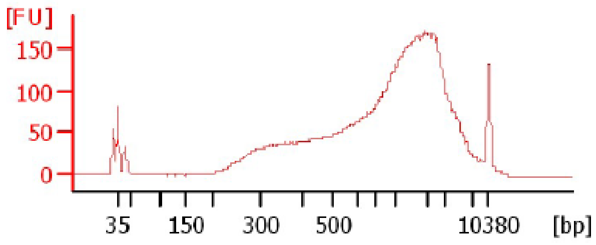

K3S1

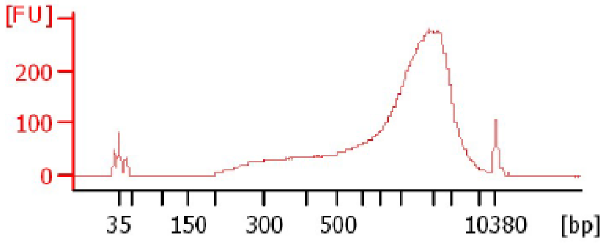

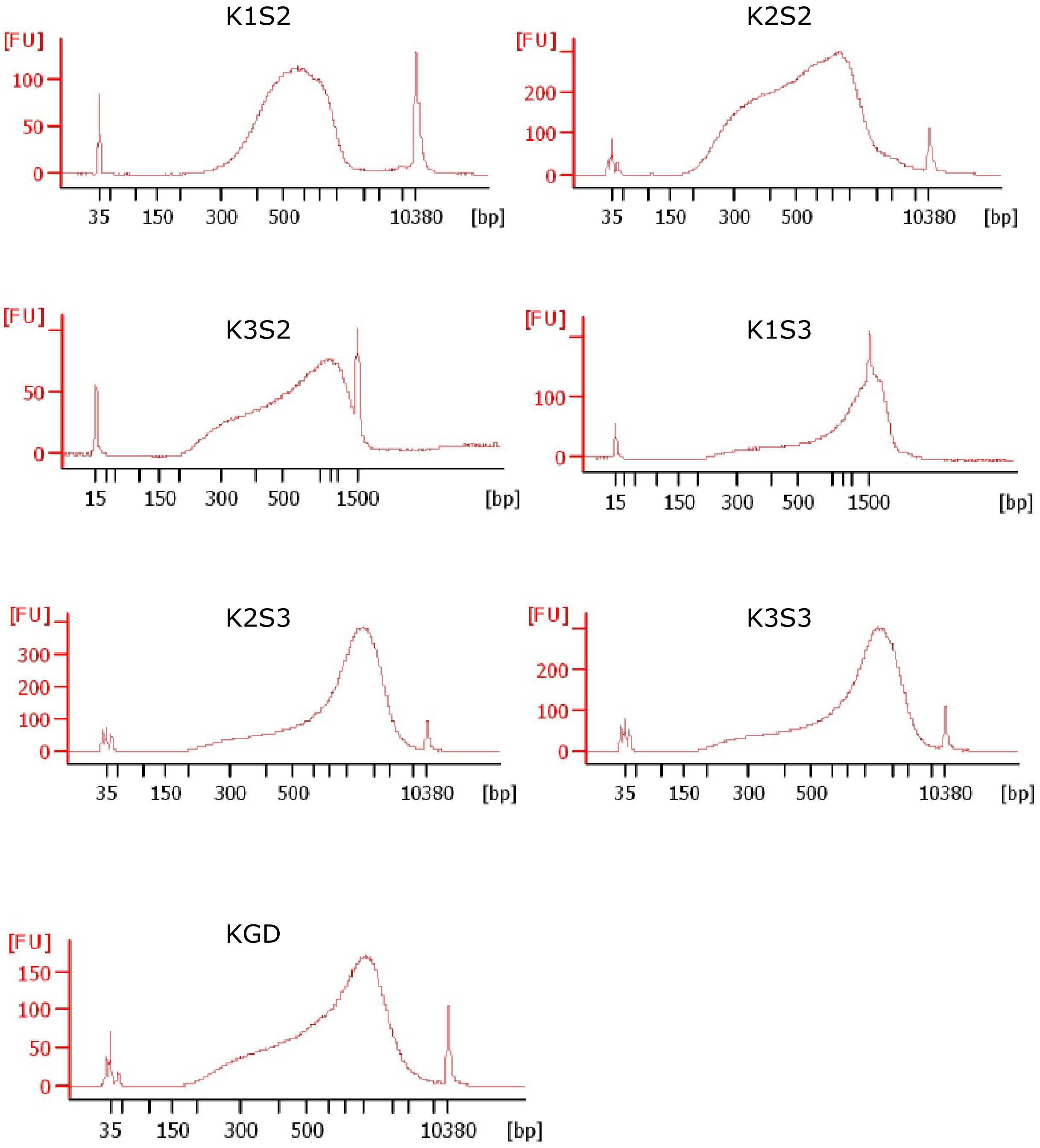
K1S2
[FU]
100
50
0
35
150
300
500
10380
[bp]
K2S2
[FU]
200
100
0
35
150
300
500
10380
[bp]
K3S2
[FU]
50
0
15
150
300
500
1500
[bp]
K1S3
[FU]
100
0
15
150
300
500
1500
[bp]
K2S3
[FU]
300
200
100
0
35
150
300
500
10380
[bp]
K3S3
[FU]
200
100
0
35
150
300
500
10380
[bp]
KGD
[FU]
150
100
50
0
35
150
300
500
10380
[bp]

Quality statistics for DNA library preparation with Nextera

SAMPLE	LIBRARY ID	QUBIT		AGILENT
		ng/ML	ng/ul	Average size (bp)
NGD	Nextera	122	24,4	605
N2BL	Nextera	131	26,2	595
N3BL	Nextera	125	25	599
N1S1	Nextera	140	28	587
N2S1	Nextera	102	20,4	580
N3S1	Nextera	124	24,8	609
N1S2	Nextera	139	27,8	578
N2S2	Nextera	113	22,6	600
N3S2	Nextera	139	27,8	591
N1S3	Nextera	128	25,6	604
N2S3	Nextera	107	21,4	602
N3S3	Nextera	126	25,2	574
N1BL	Nextera	105	21	664

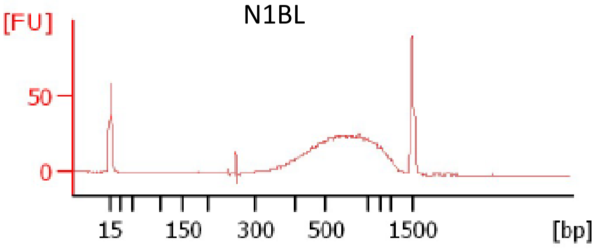

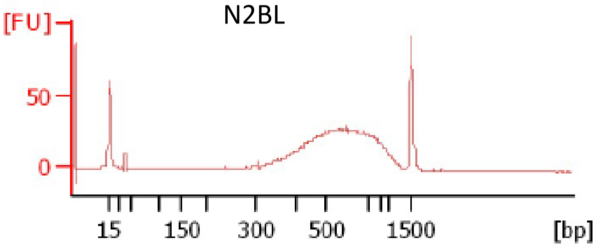

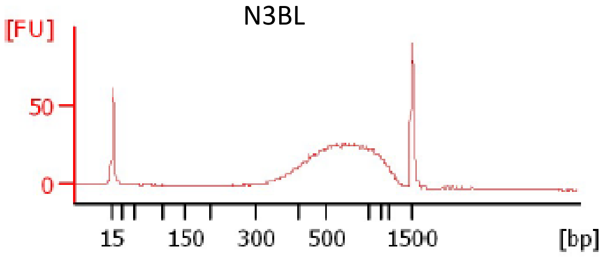

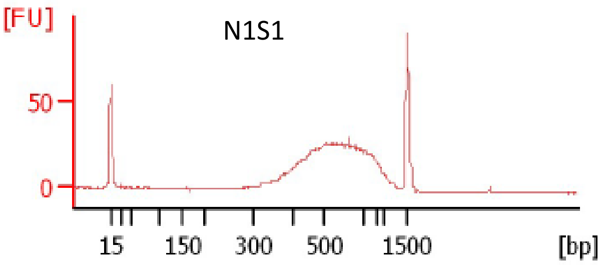

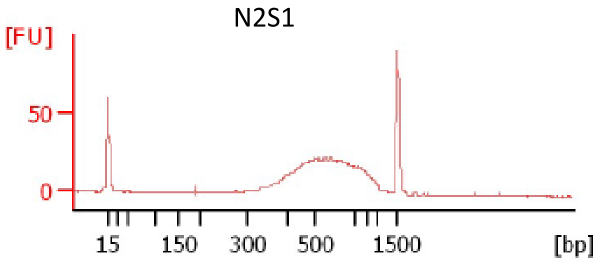

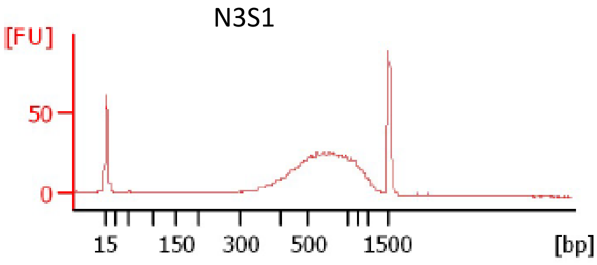

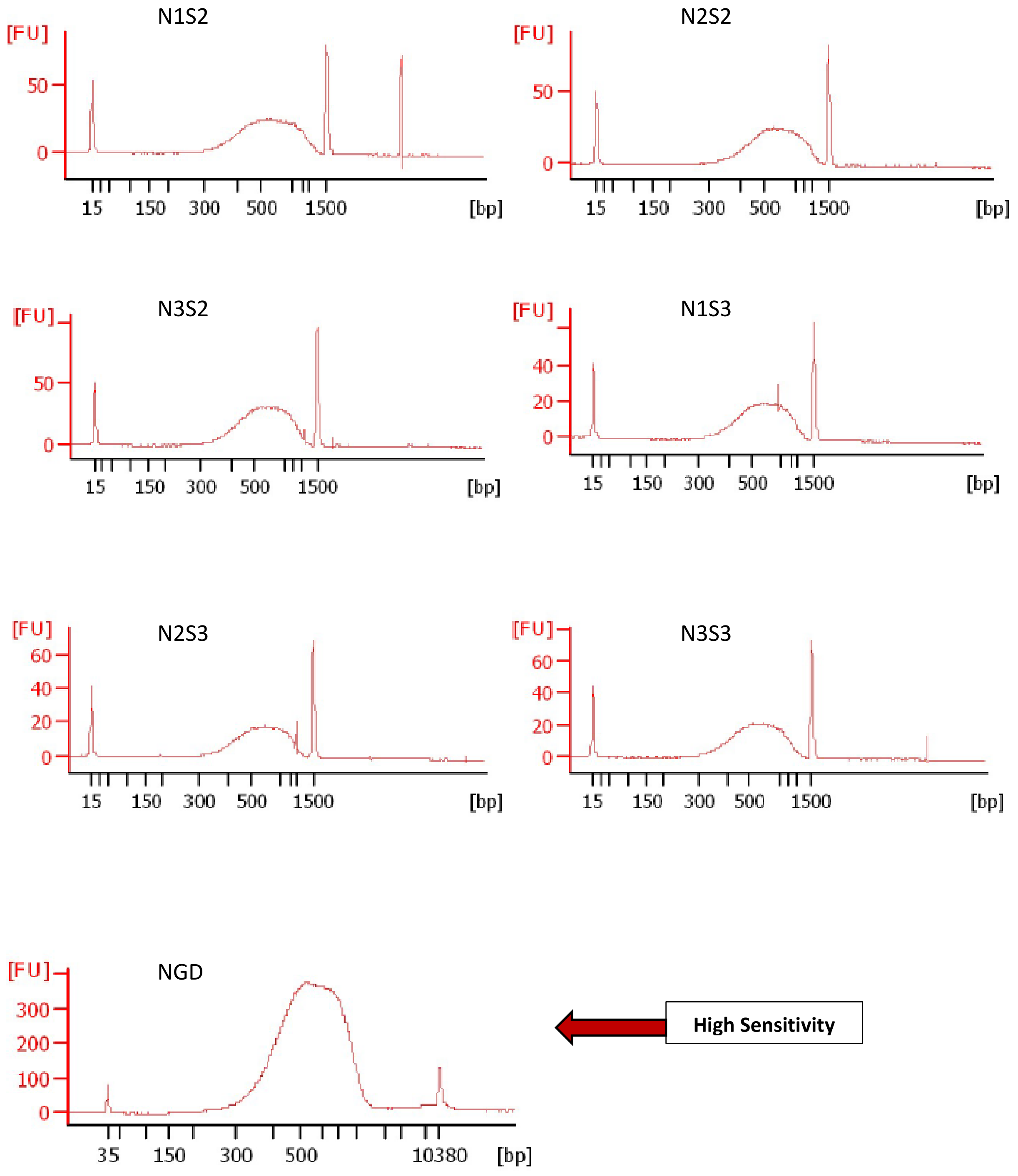
N1S2
[FU]
50
0
15
150
300
500
1500
[bp]
N2S2
[FU]
50
0
15
150
300
500
1500
[bp]
N3S2
[FU]
50
0
15
150
300
500
1500
[bp]
N1S3
[FU]
40
20
0
15
150
300
500
1500
[bp]
N2S3
[FU]
60
40
20
0
15
150
300
500
1500
[bp]
N3S3
[FU]
60
40
20
0
15
150
300
500
1500
[bp]
NGD
[FU]
300
200
100
0
35
150
300
500
10380
[bp]
High Sensitivity

Quality statistics for DNA library preparation with Qiagen

SAMPLE	LIBRARY ID	QUBIT		AGILENT
		ng/ML	ng/ul	Average size (bp)
Q1BL	QIAGEN	313	62,6	544
Q2BL	QIAGEN	251	50,2	549
Q3BL	QIAGEN	346	69,2	668
Q1S1	QIAGEN	275	55	396
Q2S1	QIAGEN	296	59,2	464
Q3S1	QIAGEN	317	63,4	428
Q1S2	QIAGEN	288	57,6	402
Q2S2	QIAGEN	301	60,2	420
Q3S2	QIAGEN	253	50,6	416
Q1S3	QIAGEN	210	42	396
Q2S3	QIAGEN	262	52,4	406
Q3S3	QIAGEN	283	56,6	400
QGD	QIAGEN	174	34,8	961

Q1BL

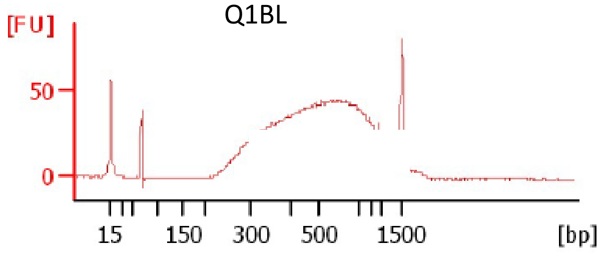

Q2BL

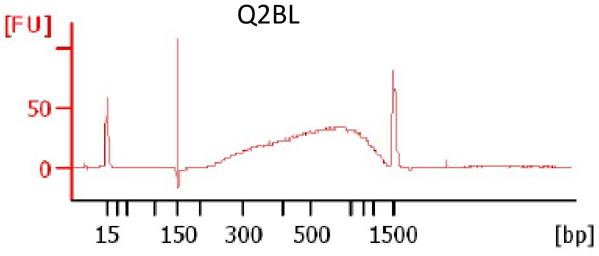

Q3BL

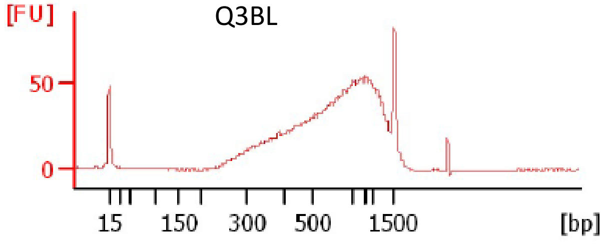

Q1S1

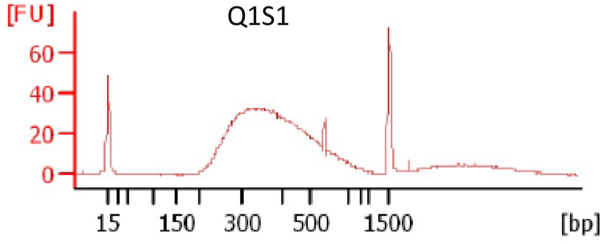

Q2S1

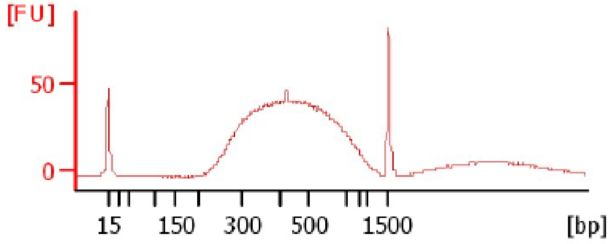

Q3S1

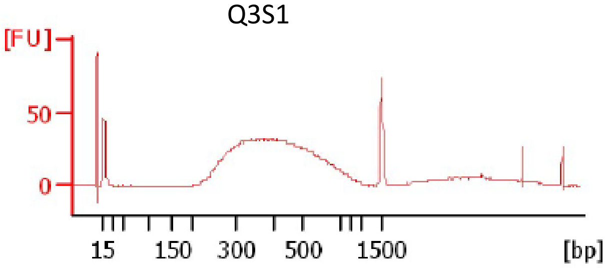

Q1S2

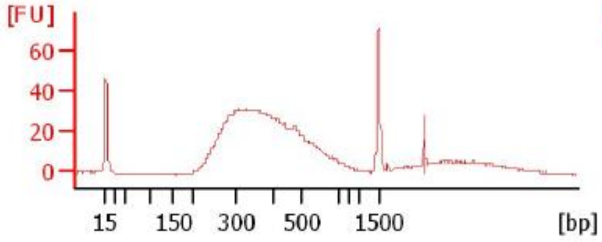

Q2S2

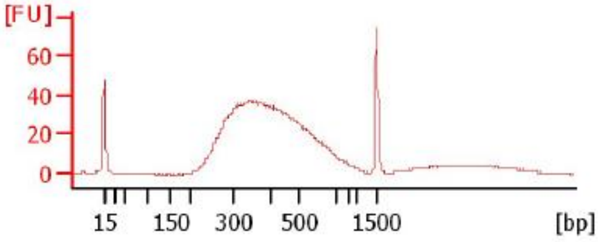

Q3S2

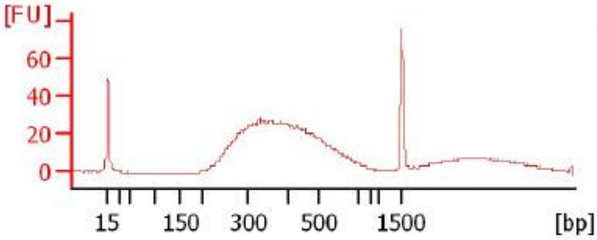

Q1S3

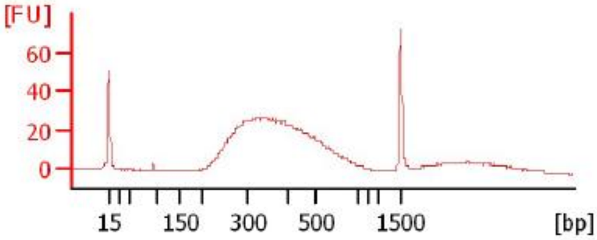

Q2S3

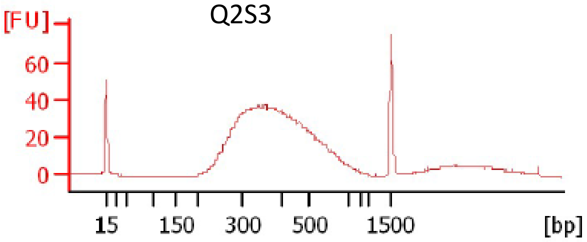

Q3S3

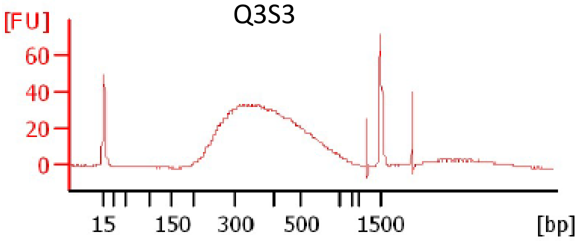

QGD

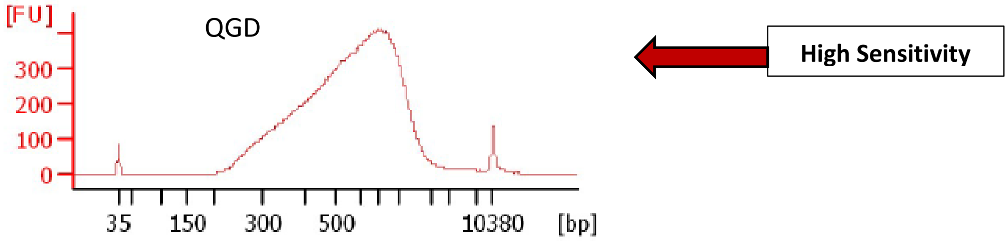

Supplement: Supplementary file 2 — Supplementary Information 2. [file 41598_2022_12037_MOESM2_ESM.zip › Supplementary files/Supplementary File 1.pdf]
