# Supplementary figures and images for: The standardisation of the approach to metagenomic human gut analysis: from sample collection to microbiome profiling
Source: Sci Rep. 2022 May 19;12:8470. doi: 10.1038/s41598-022-12037-3 (PMC9120454; doi:10.1038/s41598-022-12037-3)

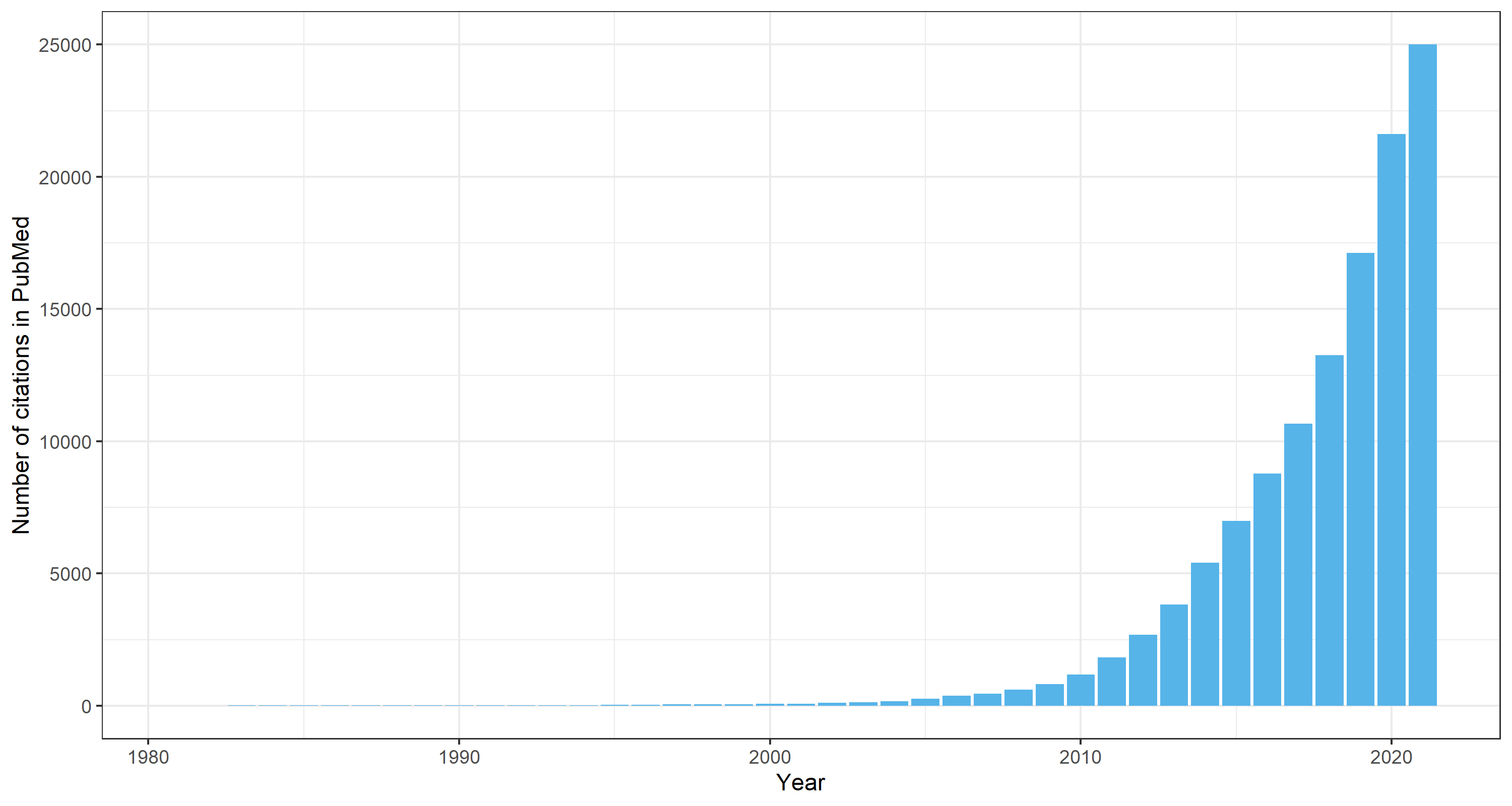

Supplement: Supplementary file 2 — Supplementary Information 2. [file 41598_2022_12037_MOESM2_ESM.zip › Supplementary files/Supplementary Figure 1.png]

# Abundance distribution for correctly identified species

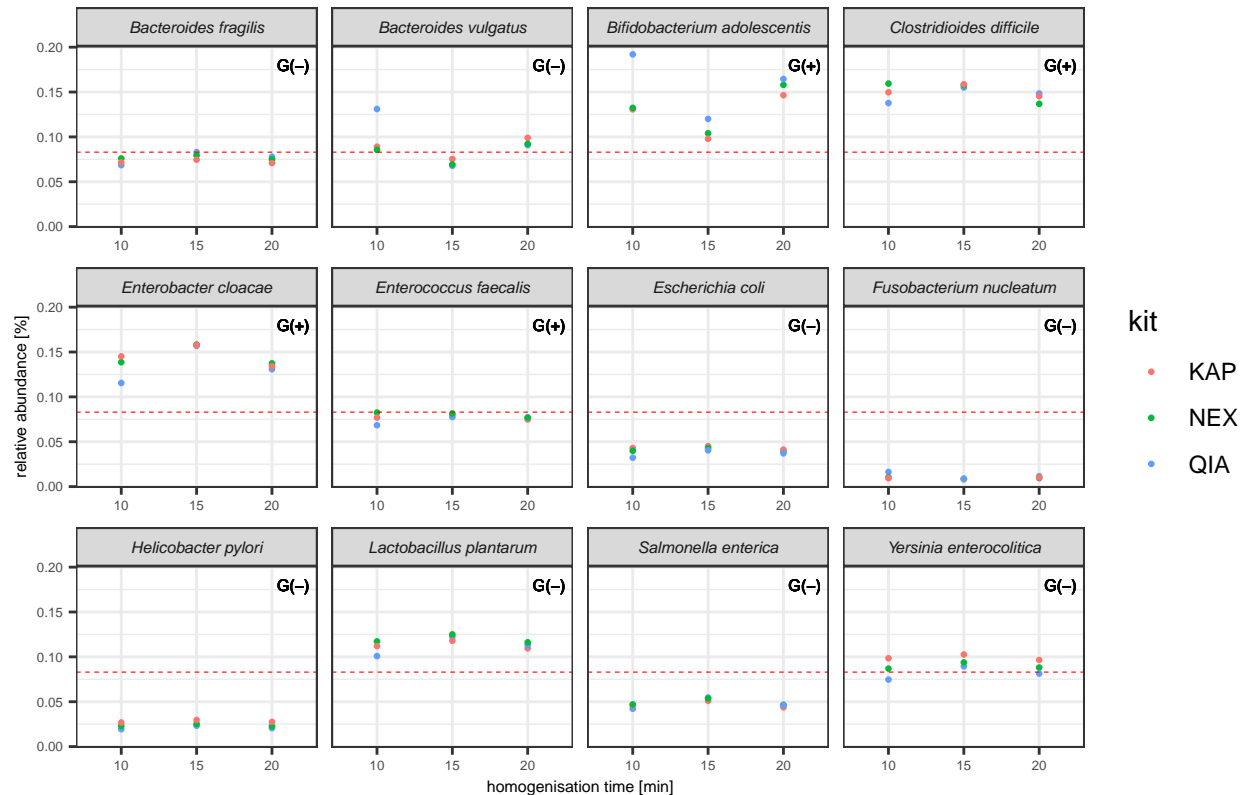

Supplement: Supplementary file 2 — Supplementary Information 2. [file 41598_2022_12037_MOESM2_ESM.zip › Supplementary files/Supplementary Figure 12.pdf]

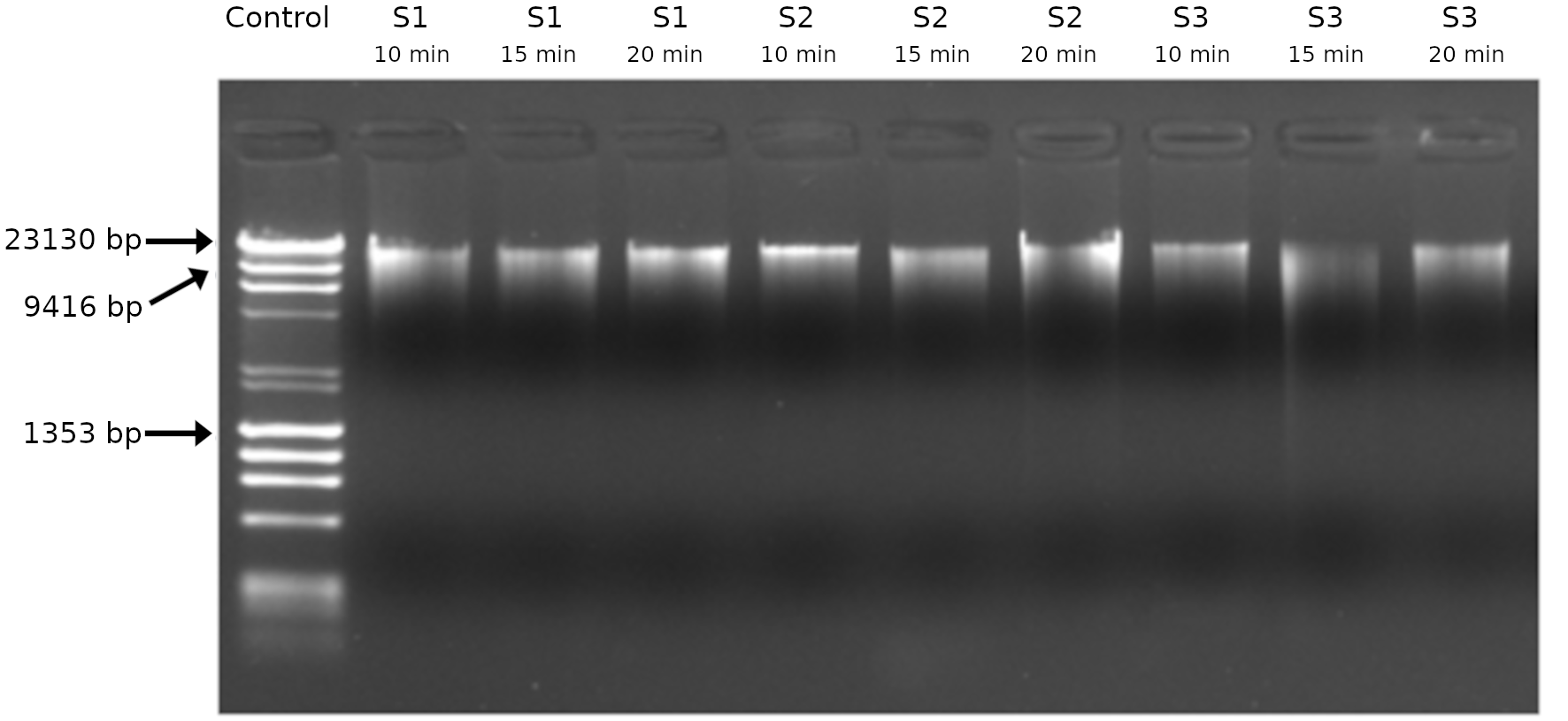

Supplement: Supplementary file 2 — Supplementary Information 2. [file 41598_2022_12037_MOESM2_ESM.zip › Supplementary files/Supplementary Figure 2.png]
